# Global transcriptional control by glucose and carbon regulator CcpA in *Clostridium difficile*

**DOI:** 10.1093/nar/gks864

**Published:** 2012-09-18

**Authors:** Ana Antunes, Emilie Camiade, Marc Monot, Emmanuelle Courtois, Frédéric Barbut, Natalia V. Sernova, Dmitry A. Rodionov, Isabelle Martin-Verstraete, Bruno Dupuy

**Affiliations:** ^1^Laboratoire Pathogenèse des Bactéries Anaérobies, Département de Microbiologie Institut Pasteur, ^2^Université Paris Diderot, Sorbonne Paris Cité, Cellule Pasteur, 25 rue du Docteur Roux, Paris 75015, France, ^3^Institute for Information Transmission Problems, Russian Academy of Sciences, Moscow 127994, Russia and ^4^Sanford-Burnham Medical Research Institute, La Jolla, CA 92037, USA

## Abstract

The catabolite control protein CcpA is a pleiotropic regulator that mediates the global transcriptional response to rapidly catabolizable carbohydrates, like glucose in Gram-positive bacteria. By whole transcriptome analyses, we characterized glucose-dependent and CcpA-dependent gene regulation in *Clostridium difficile*. About 18% of all *C. difficile* genes are regulated by glucose, for which 50% depend on CcpA for regulation. The CcpA regulon comprises genes involved in sugar uptake, fermentation and amino acids metabolism, confirming the role of CcpA as a link between carbon and nitrogen pathways. Using combination of chromatin immunoprecipitation and genome sequence analysis, we detected 55 CcpA binding sites corresponding to ∼140 genes directly controlled by CcpA. We defined the *C. difficile* CcpA consensus binding site (*cre_CD_* motif), that is, ‘RRGAAAANGTTTTCWW’. Binding of purified CcpA protein to 19 target *cre_CD_* sites was demonstrated by electrophoretic mobility shift assay. CcpA also directly represses key factors in early steps of sporulation (Spo0A and SigF). Furthermore, the *C. difficile* toxin genes (*tcdA* and *tcdB*) and their regulators (*tcdR* and *tcdC*) are direct CcpA targets. Finally, CcpA controls a complex and extended regulatory network through the modulation of a large set of regulators.

## INTRODUCTION

*Clostridium difficile*, a Gram-positive, anaerobic, spore forming bacterium, is an emerging pathogen. It is the leading cause of antibiotic-associated diarrhoea and is implicated in almost all cases of pseudomembranous colitis in humans. A higher morbidity and mortality rate because of *C. difficile* has been described in recent years (1,2). The development of *C. difficile* infection (CDI) is usually associated with antibiotic treatment, which disrupts the gut flora, allowing this bacterium to colonize the intestine (3). The spectrum of diseases caused by *C. difficile* is highly variable and depends not only on host factors but also, for the severe forms, on the level of toxin production (4).

The pathology process of *C. difficile* is divided in three main steps as follows: spore germination, host colonization and toxin production. After host infection by *C. difficile* spores, germination occurs, and the vegetative forms multiply in the gut. *Clostridium difficile* can adhere to the mucus layer and colonize the human gut (5). Pathogenic strains then produce two toxins (TcdA and TcdB), which are considered as the major virulence factors, that lead to the disruption of the actin cytoskeleton of intestinal epithelium cells (6,7), therefore, conferring the CDI symptoms (diarrhoea, epithelial apoptosis and ulceration). The toxin genes lie within a 19.6-kb pathogenicity locus (PaLoc) that also includes three accessory genes encoding the specific transcriptional regulators of toxin genes (TcdR and TcdC), and a holin-like pore forming protein (TcdE) involved in toxin secretion (8–10).

Among the other potential virulence factors, *Clostridium difficile* binary toxin (CDT), which is produced by 35% of *C. difficile* strains, has been recently shown to play a role in adherence to the host intestinal epithelial cells (11). Other factors, such as the adhesin Cwp66, the flagellum, the fibronectin-binding protein FbpA and the surface layer protein SlpA also play a role in cell adherence [for review see reference (5)]. *Clostridium difficile* also displays some surface proteolytic activities that seem to contribute to nutrients release (12), such as the cysteine protease Cwp84, which is also involved in SlpA maturation (13). Nevertheless, *in vivo* virulence processes of CDI are still poorly understood.

Many bacterial pathogens produce virulence factors to overcome the drastic changes in environment that they encounter during infection. Consequently, the regulation of the virulence genes is an important step of the pathogenicity process. In *C. difficile,* toxin production increases as cells enter into stationary phase and is modulated in response to different environmental signals, such as temperature, biotin limitation, the presence of antibiotics, of butyric acid or butanol and of certain amino acids like cysteine or proline (14). This indicates a correlation between environmental signals and virulence factors regulation. The transcription of *tcdA* and *tcdB* is also repressed by the presence of rapidly metabolizable carbohydrates, such as glucose or other sugars taken up by the phosphoenolpyruvate-dependent phosphotransferase system (PTS) (15). The PTS is composed of enzyme I and histidine-containing phosphocarrier protein (HPr), present in the cytoplasm, and the carbohydrate specific transporter enzyme II, consisting usually of three domains (EIIA, EIIB and EIIC). The carbon catabolite repression (CCR) in bacteria is generally regarded as a regulatory mechanism to ensure sequential utilization of carbohydrates (16). CCR has been extensively studied in *Bacillus subtilis* [for review see references (16,17)]. The global mechanism of CCR is mediated by the pleiotropic regulator CcpA, a member of the LacI/GalR family of transcription factors (16). CcpA represses the transcription of several hundred catabolic genes and activates the expression of some genes of overflow metabolism (17). Positive and negative transcriptional regulation of CcpA-controlled genes involves the binding of CcpA to the *cis*-acting catabolite responsive element named *cre* site. In *Bacilli*, the DNA-binding activity of CcpA is enhanced by its interaction with HPr-Ser-P, a phosphorylated form of HPr (16). Although CcpA controls most genes subjected to catabolite control in *B. subtilis*, transcriptomic data have also revealed the existence of CcpA-independent CCR mechanisms (18,19).

CcpA plays a central role in the control of metabolism in several Firmicutes, such as *Lactococcus lactis*, *Bacillus cereus*, *Clostridium acetobutylicum* and several streptococci and staphylococci (20–24). CcpA also regulates the expression of virulence-associated genes. In *Staphylococcus aureus*, *Streptococcus pneumoniae*, *Clostridium perfringens* and *Bacillus anthracis*, CcpA participates in the control of toxin production, colonization, capsule synthesis, biofilm formation and/or antibiotic resistance (25–29). Moreover, *ccpA* mutants of *S. aureus* and *B. anthracis* are attenuated on virulence in animal models of infection (24,28). The coordinate CcpA-dependent regulation of virulence and carbon utilization genes might be critical for fitness when pathogens compete with other microbes for niche colonization (23).

Recently, we have demonstrated that CcpA is involved in the glucose-dependent repression of *C. difficile* toxin production. This repression is because of a direct binding of CcpA to the regulatory region of the *tcdA* and *tcdB* genes (30). This is the first evidence that CcpA acts as a link between carbon source utilization and virulence gene expression in *C. difficile*. This bacterium has a large repertoire of genes dedicated to carbohydrate catabolism. *Clostridium difficile* ability to use an extended range of carbohydrates might be important during the pathogenesis process by promoting survival and growth in the intestine (31). Moreover, other factors important for infection and pathogenesis (besides the two major toxins) may be under CCR. The aim of this study was to analyse the global impact of glucose on gene expression and the regulatory role of CcpA in *C. difficile.* To define the processes coordinately controlled by glucose mediated or not by CcpA, we performed a transcriptome analysis of JIR8094 and its isogenic *ccpA* mutant strains grown in the presence or absence of glucose. We further combined a genome scale *in vivo* determination of CcpA direct targets to an *in silico* analysis and an *in vitro* protein-DNA interaction study to define the CcpA DNA-binding motif, therefore, characterizing the CcpA regulon in *C. difficile*. The transcriptomic data were also correlated with phenotypic tests concerning the impact of glucose addition and CcpA inactivation on the fermentation process and sporulation efficiency. The data obtained in this study provide new insights into understanding the links between nutrient acquisition and virulence in *C. difficile*.

## MATERIALS AND METHODS

### Bacterial strains and growth conditions

The *C. difficile* strain JIR8094 (32) and its isogenic Δ*ccpA* mutant (30) were grown anaerobically (5% H_2_, 5% CO_2_ and 90% N_2_) in tryptone–yeast extract (TY) or TY medium containing 0.5% glucose (TYG) in Freter chamber (15). Sporulation medium (SM) (33) was used for sporulation assays. SM medium contained per liter: 90 g Bacto–tryptone, 5 g Bacto–peptone, 1 g (NH_4_)_2_SO_4_, 1.5 g Tris base and pH at 7.4*.* When necessary, cefoxitin (25 µg/ml) or erythromycin (2.5 µg/ml) was added to *C. difficile* cultures.

### Sporulation assay

Mid-exponential growth cultures of *C. difficile* JIR8094 and its isogenic *ccpA* mutant in SM medium were used to inoculate 5 ml of SM broth or SM broth supplemented with 0.5% glucose. After 24, 48 and 96 h of growth, 350 µl of culture was divided into two samples. To determine the number of colony forming unit (CFU), one sample was serially diluted and plated on Brain–Heart Infusion (BHI) with 0.1% taurocholate (Sigma). To determine the number of spores in the culture, the other sample was heated at 60°C for 30 min before plating in BHI with 0.1% taurocholate. The percentage of sporulation was determined as the ratio of the number of spores/ml and the number of bacteria per millilitre.

### Volatile fatty acid analysis

The end products of bacteria fermentation were detected by gas–liquid chromatography. Strain JIR8094 and the *ccpA* mutant were grown in TY or TYG for 48 h at 37°C. After centrifugation, supernatants were recovered and mixed with sulphuric acid and ether to extract the volatile fatty acids. For each sample, 5 µl of the supernatants was injected in a gas chromatograph (CP-3380; Varian) equipped with a flame ionization detector and connected to an integrator (model C-55A; Shimadzu). A glass column (2 m × 4 mm) packed with 10% SP 1000 plus 1% H_3_PO_4_ on Chromosorb W AW (100/120 mesh) was used. The instrument was operated at 170°C for 18 min. The operating conditions were as follows: injector temperature, 200°C; detector temperature, 200°C; carrier gas (nitrogen) flow rate, 30 ml min^−^^1^. Each peak of the gas–liquid chromatography patterns was identified by the retention time compared with that obtained with standard (2-methylpentanoic acid at 10 mM). The amounts of fatty acids were calculated by comparison with an internal standard (34).

### Microarray design, DNA-array hybridization

The microarray of *C. difficile* 630 genome was designed as previously described (35) [Gene Expression Omnibus (GEO) database accession number GPL10556]. Transcriptome was performed using four different RNA preparations for each condition (JIR8094 in TY and TYG, *ccpA* mutant in TY and TYG at 10 h of growth). Labelled DNA hybridization to microarrays and array scanning were done according to Saujet *et al.* (35). The complete experimental data set was deposited in the GEO database with a superseries record accession number GSE35152.

### Chromatin immunoprecipitation followed by microarray hybridization (ChIP-on-Chip) experiments

The *C. difficile* strain 630 genome was obtained from the European Molecular Biology Laboratory (EMBL) database. Probe design for the microarray was performed by using OligoArray 2.0 software (36). One to three oligonucleotides were designed for each 3774 intergenic region, including 100 bp of the 3′ and 5′ ends of genes edge. We could not design oligonucleotides for 123 intergenic regions. Probes were replicated twice on the Agilent produced microarrays to reach a final density of 15 200 probes per array. A total of 459 positive controls and 77 negative controls were also included. The description of the microarray design was submitted to the GEO database (accession number GPL15104).

The chromatin immunoprecipitations (ChIP) were performed using three independent cultures for each strain. The *C. difficile* JIR8094 and its isogenic *ccpA* mutant (negative control) strains were grown in TYG medium until an optic density (OD)_600_ of 0.6 (37). The cells were treated with formaldehyde (final concentration: 1%) and incubated for 10 min at 37°C. Cross-linking was quenched by addition of glycine (final concentration: 400 mM). Cells were then collected by centrifugation, washed twice with Tris-buffered saline (20 mM Tris–HCl pH 7.5, 150 mM NaCl) and stored at −80°C. Pellets were resuspended in 10 ml of buffer A [50 mM 4-(2-hydroxyethyl)-1-piperazineethanesulfonic acid (HEPES)–KOH pH 7.5, 150 mM NaCl, 1 mM ethylenediaminetetraacetic acid (EDTA), 1% Triton X-100, 0.1% sodium dodecyl sulphate (SDS), 0.1% sodium deoxycholate and Complete antiprotease] and sonicated in a water bath sonicator (Bioruptor, Diagenode) to shear DNA to an average size of 400–600 bp. After centrifugation, the supernatant was incubated for 3 h at 4°C in the presence of 40 µl of Dynabeads^®^ Pan mouse IgG (Life Technologies^™^) on a rotating wheel. Dynabeads were then removed (non-specific immunoprecipitation), and supernatant was incubated at 4°C overnight with polyclonal anti-CcpA antibodies (1/500 dilution). The mixture was then incubated with 50 µl of Dynabeads^®^ Pan mouse IgG (Life Technologies^™^) for 90 min at 4°C and washed twice with buffer A, once with buffer A plus 500 mM NaCl, once with buffer B (10 mM Tris–HCl pH 8.0, 1 mM EDTA, 0.1% Nonidet-P40,0.5% sodium deoxycholate) and once with 10 mM Tris–HCl pH 7.5, 1 mM EDTA. Antibodies bound to the magnetic beads were then eluted for 20 min at 65°C with 100 µl of elution buffer (50 mM Tris–HCl pH 7.5, 10 mM EDTA, 1% SDS). Samples were then treated with proteinase K (50 µg) for 2 h at 56°C and 6 h at 65°C in a 0.5× elution buffer. Immunoprecipitated DNA samples were finally purified using the QIAquick polymerase chain reaction (PCR) purification kit (QIAGEN). DNA obtained from immunoprecipitation was then labelled with Cy5 or Cy3 using the Bioprime plus array CGH indirect genomic labelling system (Invitrogen) as recommended by the manufacturer. Labelled DNA hybridization to microarrays and array scanning were done according to Saujet *et al.* (35). The complete experimental data set were deposited in the GEO database with the accession number GSE34971.

### Microarrays or ChIP-on-Chip data analysis

All the slides, from microarray or ChIP-on-Chip experiment, were analysed using R and linear model for microarray data software from Bioconductor project (www.bioconductor.org). For each slide, we corrected background with the ‘normexp’ method (38), resulting in strictly positive values and reducing variability in the log ratios for genes with low levels of hybridization signal. Then, we normalized each slide with the ‘loess’ method (39). To test for differential expression, we used the Bayesian adjusted *t*-statistics, and we performed a multiple testing correction of Benjamini and Hochberg (40) based on the false discovery rate. For the transcriptome data, a gene is considered as differentially expressed when the *P*-value is <0.05. For the ChIP-on-Chip experiment, a DNA region is considered as significantly enriched with a *P*-value <0.05. The enrichment factor for a given intergenic region was calculated as the ratio of hybridization of immunoprecipitated DNA in the JIR8094 compared with the *ccpA* mutant strains.

### Bioinformatic analysis of CcpA binding regions

For the prediction of CcpA binding sites in *C. difficile* genome, we searched for a common motif in the promoter region of 20 direct target genes identified in ChIP-on-Chip experiments with a high ratio of enrichment (CD0201, CD0279A, CD0395, CD0576, CD0833 CD1477, CD1536, CD1768, CD1893, CD2241, CD2280, CD2327, CD2347, CD2600, CD2625, CD2702, CD3218, CD3244, CD3377, CD3664). The *de novo* motif extraction program MEME (41,42) was used for the *cre_CD_* motif identification. A positional weight matrices (PWM)-based model of the CcpA binding motif was generated and further used for genome-wide detection of additional CcpA targets in genomic upstream regions. The screening of additional CcpA binding sites upstream of the genes identified in the ChIP-on-Chip analysis and/or the CcpA transcriptomic analysis was carried out using the RegPredict web server (43). The motif was searched in the leader regions from position −250 to +50 relative to the translational start site for the MEME program and −300 and +300 for RegPredict. Scores of candidate sites were defined as the sum of positional nucleotide weights. The score threshold for the search of candidate binding sites was selected as a minimal score in the training set of known CcpA target sites (score value 4.0). The details of the identified CcpA taget binding sites and corresponding genes are captured and displayed in the RegPrecise database (regprecise.lbl.gov).

### DNA techniques and electrophoretic mobility shift assay

DNA fragments containing the putative CcpA binding site (*cre_CD_* site) of genes CD0395, CD0833, CD1477, CD1536, CD2327 and CD2600 were amplified by PCR from genomic DNA of *C. difficile* 630 strain with primers OBD713–716, OBD646–649, OBD678–681, OBD658–661, OBD706–709 and OBD664–667, respectively (Supplementary Table S1). Deletion of the *cre_CD_* site was performed on these amplified DNA fragments as described in Supplementary Figure S1. All PCR products were then used as templates to amplify and to radioactively label a smaller DNA fragment of 200–300 bp containing or not containing the putative *cre_CD_* site with the corresponding primers listed in Supplementary Table S1. The radioactively labelled DNA fragments correspond to positions −97 to +82 with respect to the translational start site of CD0395, +213 to +434 of CD0833, −146 to +75 of CD1477, +132 to +357 of CD1536, −215 to +73 of CD2327 and −183 to +22 of CD2600.

The other DNA fragments containing a *cre_CD_* site of genes CD0576 (position +44 to +300 with respect to the translational start site), CD0659 (−338 to −168 and −220 to −52), CD0664 (−18 to +205), CD0770 (−3 to +179 and +149 to +300), CD1214 (−107 to +106), CD1768 (−216 to −6), CD1893 (−193 to +30), CD1935 (−267 to −58), CD2241 (−197 to +46), CD2357 (+118 to +368), CD3218 (+197 to +405), CD3244 (−385 to −197) and CD3664 (−162 to +52) were amplified and radioactively labelled by PCR from chromosomal DNA with corresponding primers listed in Supplementary Table S1. Promoter regions of CD1893 and CD3218 and internal fragments of genes CD2327 and CD1477 without putative *cre_CD_* sites (negative controls) were also amplified and radioactively labelled by PCR from chromosomal DNA with primers OBD684–685 (position −193 to −43 with respect to the translational start site), OBD612–613 (−117 to +84), OBD710–711 (+159 to +328) and OBD704–705 (+57 to +208), respectively.

For the radioactive labelling of PCR fragments, all forward primers were end-labelled with T4 polynucleotide kinase (Fermentas and γ-^32^P- adenosine triphosphate (3000 Ci.mM^−^^1^; Perkin Elmer) as recommended by the manufacturer. After PCR, amplified labelled fragments were then purified by QIAquick® Nucleotide Removal kit (Qiagen^™^). The labelled fragments were incubated with increasing amounts of the purified CcpA–His_6_ at room temperature for 15 min in 20 µl of reaction volume containing binding buffer (10 mM Tris pH 7.5, 1 mM EDTA; 50 mM KCl; 50 μg/ml bovine serum albumin, 0.05% Nonidet P-40, 10% glycerol, 1 mM Dithiothreitol (DTT), 10 μg/ml polydI-dC) as described in Antunes *et al.* (30). Samples were loaded during electrophoresis onto a 5% non-denaturing polyacrylamide gel in Tris–glycine buffer (25 mM Tris–HCl pH 8, 190 mM glycine, 1 mM EDTA) (30). After electrophoresis, the gel was dried and analysed by autoradiography.

## RESULTS AND DISCUSSION

### Overview of the transcriptome data

The ability of bacteria to adapt to environmental changes involves a number of physiological responses and complex molecular regulatory circuits. In many pathogenic bacteria, carbohydrate substrates constitute an important signal used for compartment-specific modulation of virulence genes during infection (44). Previously, we have shown that glucose regulates *C. difficile* toxin genes expression through a direct binding of CcpA to the *tcdA* and *tcdB* regulatory regions (30), suggesting that CcpA seems to link virulence gene expression and carbohydrate availability in this bacterium. However, a comprehensive study of the role of CcpA in *C. difficile* physiology has not been undertaken so far.

To determine the role of CcpA in CCR and the global impact of glucose on gene expression, we performed a transcriptional analysis of the wild-type strain JIR8094 and its isogenic *ccpA* mutant strain grown in TY or TYG at the onset of stationary phase. Based on analysis performed (TY versus TYG in JIR8094 or in *ccpA* and *ccpA* versus JIR8094 in TYG or in TY), ∼21% of the genes (782 genes) were differentially expressed with a fold change ≥2 (Supplementary Table S2). In the presence of glucose, ∼18% of the global genome (667 genes) was differently expressed in strain JIR8094. Among them, 349 and 318 genes were up- and downregulated, respectively (Table 1). As described in *B. subtilis* and other Firmicutes, such as *B. cereus* or *Streptococcus pyogenes* (19,21,45), we observed that *C. difficile* modulates a large number of genes in response to glucose availability.

**Table 1.** Number of genes regulated by glucose and/or CcpA

Genes group^a^	Number of genes^b^
Glucose-dependent	667 (18%)
Upregulated genes	349
Downregulated genes	318
CcpA-dependent	407 (11%)
Upregulated by CcpA	197
Downregulated by CcpA	210
Regulated by glucose in a CcpA-dependent manner	361 (10%)
Upregulated genes	170
Downregulated genes	191
Regulated by glucose in a CcpA-independent manner	303 (8%)
Upregulated genes	177
Downregulated genes	126
Regulated by CcpA in absence of glucose	43 (1%)
Upregulated by CcpA	8
Downregulated by CcpA	35
Regulated by CcpA independent of glucose	3 (0.08%)
Upregulated by CcpA	2
Downregulated by CcpA	1
Atypical regulation	112 (0.3%)
Upregulated genes	39
Downregulated genes	73

Number of genes subject to regulation by glucose and/or CcpA in *in vitro* growth conditions. The percentage of genes regulated was calculated considering 3759 genes analysed in the transcriptome analysis.

^a^Groups partly overlap.

^b^A gene was considered to be regulated if transcription was induced or repressed at least 2-fold (*P* < 0.05).

The CcpA regulon consists of ∼11% of all genes (407 genes, Table 1), and these results are in accordance to what has been reported in other Firmicutes (17,20). However, only 50% of the glucose-regulated genes are controlled by CcpA in *C. difficile* in the conditions tested, whereas in *B. subtilis* >80% of the genes are controlled by CcpA in response to glucose (19). This indicates the existence of still uncharacterized signalling pathways that react to glucose in *C. difficile*. A total of 182 genes were regulated by glucose through a mechanism that is fully or partially CcpA dependent. It is worth noting that CcpA positively controls half of them in the presence of glucose. Comparable observations have been described for *S. aureus* (46). However, for most Firmicutes, the number of genes repressed by glucose in a CcpA-dependent manner largely exceeds the genes activated by CcpA (17,20,45). Finally, we noted that the expression of some genes was regulated by CcpA independently of glucose. All genes differentially expressed in response to glucose and/or controlled by CcpA are listed in Supplementary Data (Supplementary Table S2). To confirm the results obtained in the microarrays expression data, we selected a subset of 15 representative genes of different cell functions regulated by CCR and performed quantitative reverse-transcriptase (qRT)-PCR with RNA preparations from identical experimental conditions of the transcriptome. qRT-PCR results confirmed array data for majority of the tested genes (Supplementary Table S4).

In *C. difficile*, glucose and/or CcpA mainly regulate the carbohydrate and energy metabolisms. Moreover, the glucose adaptation response also implicates the regulation of genes involved in carbon sources transport, amino acids and peptide catabolism, stress response, sporulation, proteins of the cell envelope, toxin synthesis and transcriptional regulation. CcpA can control genes directly by binding in the regulatory region of the controlled genes or indirectly through other mechanisms (see later in the text). To discriminate between these two possibilities, we needed to identify the CcpA binding motif in *C. difficile*.

### Identification of direct CcpA targets by ChIP-on-Chip

In *B. subtilis*, CcpA directly controls transcription of the target genes by interacting with *cre* sites located within or downstream from the promoter regions (47). Thus, we first tried to identify the direct CcpA targets by screening the genome for the *B. subtilis cre* consensus motif (*cre_BS_*), and we then compared the list obtained with that of genes controlled by CcpA in transcriptome. However, we did not find correlation between the presence of a *cre_BS_* motif and the CcpA-dependent control. Moreover, we have previously demonstrated that the deletion of *cre_BS_*-like sites in the regulatory region of *tcdA* and *tcdB* does not abolish CcpA binding (30). This strongly suggests that the *cre* motif recognized by CcpA in *C. difficile* (*cre_CD_*) is significantly different of the *cre_BS_* motif. To identify the CcpA direct targets *in vivo* and define the *cre_CD_* consensus sequence, we performed ChIP-on-Chip experiments. For this purpose, the *C. difficile* JIR8094 strain was grown in TYG and then cross-linked with formaldehyde and further immunoprecipitated with a specific anti-CcpA antibody (30).

A total of 244 oligonucleotides were differentially enriched in the presence of CcpA with a *P*-value of <0.05 and an enrichment factor ≥1.5. We identified 55 direct targets of CcpA *in vivo* corresponding to ∼140 genes by ChIP-on-Chip experiments (Table 2). Most of these genes encode proteins involved in (i) fermentation, such as acetoacetate production (*scoB*), hydrogen production (*hymB*) and Stickland reactions (*hadA* and *prdA*); (ii) energy metabolism (CD2429A, CD1170 and CD1807); (iii) amino acids metabolism (CD1536, CD3664 and *gcvPB*), including peptidases (CD0779 and CD2347) and amino acid or peptide transporters (*oppC*, CD2173 and *brnQ*); (iv) PTS carbohydrate transporters and associated enzymes involved in sugar utilization (CD0468, CD0494, CD2280, *gatA*, *celA* and CD1336); and (v) non-PTS sugar utilization (*rbsR* and CD1586), sialic acid degradation (*nanE*) or ethanolamine transport (CD0742). Genes implicated in sporulation, such as the major response regulator Spo0A, were also directly controlled by CcpA. Moreover, genes encoding six regulatory proteins, and 12 proteins of unknown function have been identified as direct CcpA targets. Among the targets depicted by the ChIP-on-Chip experiments, 29 genes (53%) were regulated by glucose and/or CcpA in transcriptome. The overlap of genes identified in transcriptome and by ChIP-on-Chip analysis is similar to that of other studies (48,49).

**Table 2.** Direct CcpA targets identified by ChIP-on-Chip experiments

Gene	Name	FC^a^	Oligonucleotide 5′ position	Operon	*cre_CD_* motif	Position^b^	Glucose regulation^c^	CcpA regulation^c^
CD0201		11	260578	CD0201	GAGAAAATGTTTACAG	77	NR	NR
CD0279A or CD0279		7.7	340693		AAGAAAACGTTATTAC	−370	−	−
CD0298^d^	*rbsR*	1.9	359108	CD0298–CD0302	AGTAAAACGGTTTCCT	−28	−	−
CD0395^e^	*hadA*^e^	7.1	467014	CD0395–CD0401	AGGAAAACGTTATGCT	−12	−	−
CD0468		3	556433	CD0467–CD0469	TCAAAAAGATTTTCAT	−255	NR	NR
CD0494^d^		4	583410	CD0490–CD0494	AAGAAAACGTTAAGCA	26	NR	NR
CD0530^d^		4.6	635577	CD0530–CD0528	AGGAAAACGTATTATA	−78	NR	NR
CD0576^e^	*virS*^e^	33	687951		GAGAAAAGGATTTCTA	92	NR	NR
CD0580^d^	*gapN*	2.1	692989		AGAAAAACGTTAACTT	−124	NR	NR
CD0679^d^		4	821606		ATGATAACATTTTCTT	−169	NR	NR
CD0742		6.5	905449	CD0741–CD0742	GGGAAAACGATACCAA	−76	NR	NR
CD0779^d^		1.7	949162	CD0778–CD0780	GAGAAAAAGACATCAT	50	−	NR
CD0833^e^	*acnB*^e^	9.7	1009212	CD0832–CD0834	AGGAAATGGTATTTGT	284	−	−
CD0854^d^	*oppC*	5.7	1029471	CD0853–CD0856	AAGAAAACCTTATCTA	77	−	−
CD1116		36	1312695		GAGAAAAGGTTTCCAA	167	NR	+
CD1137		5.2	1336232	CD1136–CD1137	GAAAAAATGTTTTTTT	−37	NR	NR
CD1170		5.9	1368750	CD1170–CD1173	ND	ND	+	NR
CD1214^e^	*spo0A*^e^	1.5	1412498		TGTAAAAAGTTTAGTT	−42	−	−
CD1321^d^		1.6	1531839		AGGAAATAGTTAACTT	24	−	−
CD1336		1.5	1550212		TAGAAAACGTTTTAAA	−41	NR	NR
CD1385		3.6	1603687	CD1384–CD1387	AAGAAAGCGTTTTGAA	−111	−	−
CD1477^e^	*feoA*^e^	13	1714290	CD1477–CD1480	AAGATAACGATTGCTT	−52	NR	+
CD1536^e^		15.5	1781719	CD1536–*aspB*	AAGAAAACGATTTTGT	231	NR	−
CD1586		6.6	1838850	CD1584–CD1589	TAGAAAAGGTTATCAT	111	+	NR
CD1599	*thiD*	3.8	1854754	CD1599–CD1601	TTGAAAACGTTTAGTG	76	NR	–
CD1658	*gcvPB*	1.8	1925192	CD1657–CD1658	AGGAAATAGATAAGTT	−40	−	−
CD1768^d^^,^^e^		10	2045870		GAGAAAAGGTTTTGTT	−93	+	+
CD1807		3.2	2091015		ND	ND	−	−
CD1893^d^^,^^e^		6.8	2198560		TATAAAACGTTTTCTT	−75	NR	NR
CD1935^d^^,^^e^	*spoVS*^e^	1.8	2233439		AAATAAAGGTTTTCTT	−142	NR	−
CD2111^d^		6	2440328		TAGAAAATGTTTGCAG	−7	NR	NR
CD2173		1.8	2515137	CD2177–CD2172	GAGAAAAAATTATAAA	56	−	−
CD2241^d^^,^^e^	*nanE*^e^	7.8	2592979	CD2241–CD2239	AAGAAAAAGTTTTCAT	−72	NR	NR
CD2280^d^		4.5	2646506	CD2282–CD2279	AAGAAATCGTTTCTTT	100	NR	NR
CD2327^d^^,^^e^	*gatA*^e^	30	2689528	CD2327–CD2325	GGGAAAACATTTTCTT	−113	−	−
CD2344		1.7	2714126	CD2344–CD2341	AAGATAAAATTTTTTT	−152	−	−
CD2347		8	2716984		CAGAAAAGGTTTGCAA	176	+	+
CD2429A or CD2429^d^		11	2804869	CD2429A–CD2427	GAGAAAAGGTATGCAA	−9 or 217	NR	NR
CD2481^d^	*ung*	3.6	2863759	CD2482–CD2481	TTGAAAAAGATTACTA	47	NR	NR
CD2600^e^	*cstA*^e^	5.5	3009085		TGGAAATAGTTTTCTT	−67	NR	NR
CD2625^d^		4.4	3031999		AGGATAACGTTATCAT	−61	NR	NR
CD2626^d^		6	3034769	CD2627–CD2626	GAGAAAAAATATACAA	131	NR	NR
CD2678	*scoB*	15	3093914	CD2676–CD2679	ATGTAAACGTTATCTT	120	NR	NR
CD2693		1.5	3113024		AAGAAAAAAATAGGTT	4	−	−
CD2702	*brnQ*	4.5	3124254		GGGAAAACGTTAACAA	−104	−	NR
CD2879		3.8	3368263		TAGATAAGGTTTTCTT	−128	NR	NR
CD2884^d^	*celA*	1.6	3372956	CD2884–CD2880	ATGAAAAAGTTTTCTT	−186	NR	NR
CD3175^d^	*cggR*	1.5	3711205		AGGAGAACATTATCAA	109	+	−
CD3184	*dpaL*	8.5	3726740		TAGATTTGGTTTAAAT	206	NR	NR
CD3218^e^		5	3766712		GAGTTAACGTTTTCAA	254	+	−
CD3244^d^^,^^e^	*prdA*^e^	6.5	3796160	CD3244–CD3236	GAGAAAATGTTTACAA	−293	+	+
CD3377	*mgtA*	7.4	3950193	CD3377–CD3376	ND	ND	NR	+
CD3406^d^	*hymB*	3	3984409	CD3405–CD3407	CTGATAACGTTTTCTA	270	NR	NR
CD3649	*rlmH*	13	4258421		AAGAAAAGGCTTTCTA	70	NR	NR
CD3664^e^		30	4275636		AAGAAAACATTTTCAT	−30	NR	NR

^a^Fold Change (FC) corresponding to enrichment factor in ChIP-on-Chip experiments.

^b^*cre_CD_* motif position from ATG start codon. ND = not detected.

^c^Gene regulation resulting from transcriptome and qRT-PCR analysis. ‘–’ for downregulation, ‘+’ for upregulation and ‘NR’ for absence of regulation. For more details, see Supplementary Table S2.

^d^Indicates the existence of a second putative *cre_CD_* motif.

^e^*cre_CD_* sites confirmed by EMSA experiments.

### *In silico* definition of a *cre_CD_* consensus

The region of *C. difficile* genes determined as direct CcpA targets by ChIP-on-Chip experiments with an enrichment factor >5 was searched for common CcpA binding sites to define the *cre_CD_* motif using the MEME software. A conserved 16-bp pseudopalindromic motif (RRGAAAASGTTTTCWT, where R stands for adenine or guanine, S stands for guanine or cytosine and W stands for adenine or thymine) was identified in the upstream regions of 12 CcpA target genes. Then, we used this motif and the RegPredict web server (43) to search for similar CcpA binding sites for all genes identified by the ChIP-on-Chip experiment. Among the 55 CcpA direct target genes, we failed to identify a *cre_CD_* motif for only three genes (CD1170, CD1807, CD3377), despite the fact that CD1807 and CD3377 were controlled by CcpA in transcriptome (Table 2). This could be explained by a degeneration of their binding sites as proposed for some targets of the *Escherichia coli* NsrR repressor identified *in vivo* using ChIP-on-Chip technology (48).

**Figure 1.**
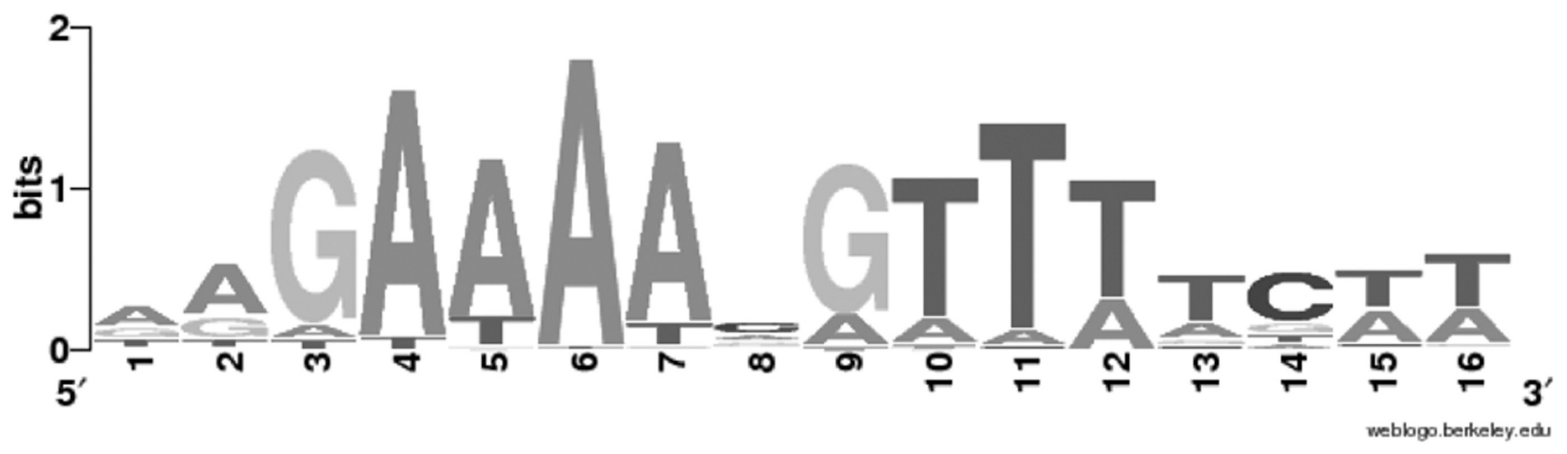
*C. difficile cre_CD_* consensus of the CcpA binding motif. The motif sequence logo was created based on the alignment of all the proposed direct targets of CcpA listed in Table 2, using the weblogo website (http://weblogo.berkeley.edu). The height of the letters is proportional to their frequency.

Interestingly, for some target sites that reside inside of operons, we observed a clear correlation between the position of the oligonucleotides bound by CcpA in ChIP-on-Chip and the location of a putative *cre_CD_* site. This is the case for *acnB, oppC,* CD1385*, gcvPB, scoB,* CD2626 and *hymB* (Table 2). The alignment of all probable CcpA binding motifs listed in Table 2 allows the establishment of a *cre_CD_* consensus (Figure 1). The *cre_CD_* motif identified in this work for *C. difficile* has limited similarity with the *cre* consensus previously identified in various bacteria from the bacilli class of Firmicutes including *Bacillus*, *Lactococcus*, *Enterococcus* and *Streptococcus* species (20,21,47,50). Besides the guanine and the cytosine at 3rd and 14th positions, respectively, which are identical in both motifs, the *cre_CD_* consensus is not similar to the *cre_BS_* motif (Figure 1). Importantly, the *cre_CD_* motif lacks the essential central CG bases (16) present in the *cre_BS_* consensus (WTGNNARCGNWWWCAW). These observations correlate with the weak similarities of CcpA helix-turn-helix (HTH)-DNA binding domain from these bacteria (47% identity), whereas, for instance, the HTH motif of CcpA of *L. lactis* has 70% identity with that of *B. subtilis*, and both have similar *cre* sites.

### CcpA directly binds to the *cre_CD_* motif of genes identified by ChIP-on-Chip

**Figure 2.**
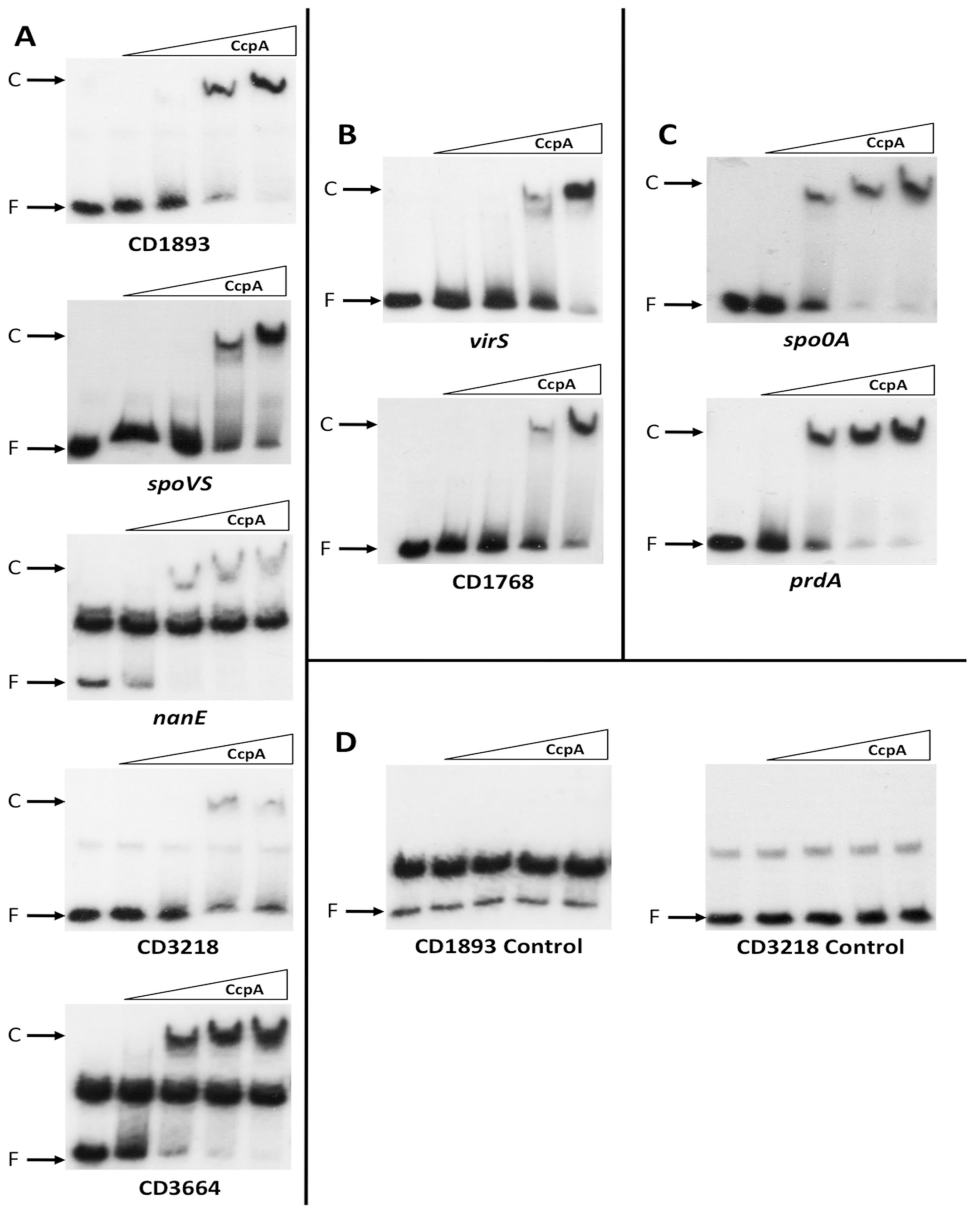
Binding of CcpA to different targets with a putative *cre_CD_* sequence identified by ChIP-on-Chip experiments. EMSAs of DNA fragments containing the *cre_CD_* binding sites of the CD1893, *spoVS* (CD1935), *nanE* (CD2241), CD3218, CD3664, *virS* (CD0576), CD1768, *spo0A* (CD1214) and *prdA* (CD3244) genes. The ^32^P-labelled DNA fragments were incubated with increasing concentrations of purified CcpA–His_6_ as described in the ‘Materials and Methods’ section: (**A**) 0, 32, 65, 97 and 129 nM of CcpA–His_6_; (**B**) 0, 97, 129, 194 and 259 nM of CcpA–His_6_; (**C**) 0, 129, 259, 388 and 518 nM of CcpA–His_6_. (**D**) Negative controls corresponding to the DNA fragments containing promoter regions of CD1893 and CD3218 without putative *cre_CD_* sites were incubated with 0, 65, 97 and 129 nM of CcpA–His_6_, and 0, 97, 129, 194 and 259 nM of CcpA–His_6_, respectively. The autoradiograms of the gels are shown. F = free DNA; C = protein–DNA complex.

**Figure 3.**
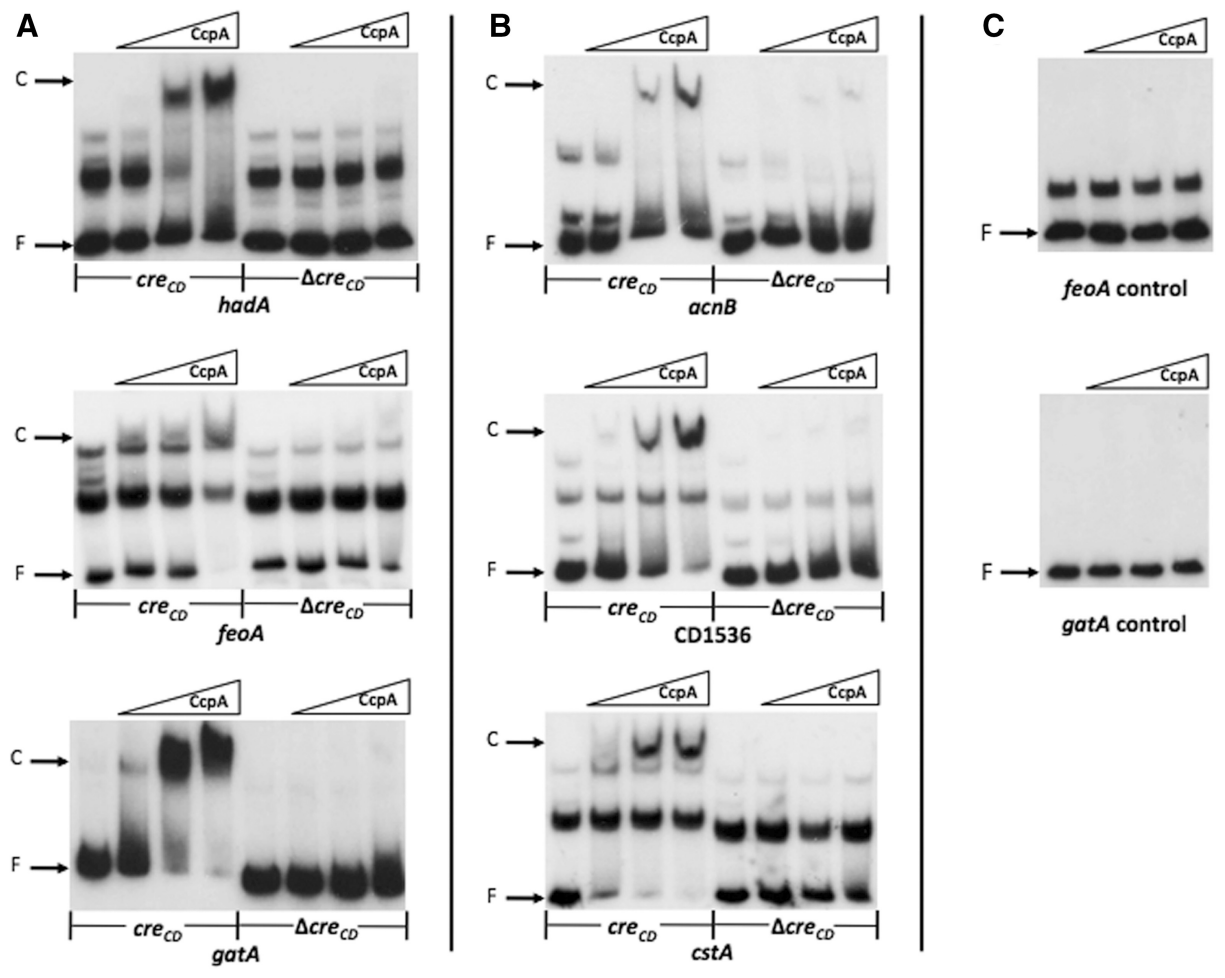
Impact of *cre_CD_* deletion on the binding of CcpA to the *hadA*, *acnB*, *feoA*, CD1536, *gatA* and *cstA* target genes. EMSAs of DNA fragments containing the *cre_CD_* binding site (*cre_CD_*) or the same DNA fragment where the *cre_CD_* site has been deleted (Δ*cre_CD_*) of the *hadA* (CD0395), *feoA* (CD1477), *gatA* (CD2327), *acnB* (CD0833), CD1536 and *cstA* (CD2600) genes. The ^32^P-labelled DNA fragments were incubated with increasing concentrations of purified CcpA–His_6_ as described in the ‘Materials and Methods’ sections: (**A**) 0, 13, 32 and 65 nM of CcpA–His_6_; (**B**) 0, 65, 97 and 129 nM of CcpA–His_6_. (**C**) Internal ^32^P-labelled DNA fragments of *feoA* and *gatA* genes without putative *cre_CD_* sites were incubated with 0, 65, 97 and 129 nM of CcpA–His_6_ (negative controls). The autoradiograms of the gels are shown. F: free DNA; C: protein–DNA complex.

To validate the ChIP-on-Chip experiment and confirm the role of the *cre_CD_* site in the CcpA binding activity, electrophoretic mobility shift assays (EMSAs) were performed using purified *C. difficile* CcpA_His6_ (30) and DNA fragments containing a putative *cre_CD_* site of 15 CcpA targets (Table 2). The CcpA transcriptional regulator induced a shift in mobility of DNA fragments containing a *cre_CD_* site of all genes tested (CD1893, *spoVS*, *nanE*, CD3218, CD3664, *virS*, CD1768, *spo0A*, *prdA, hadA*, *feoA*, *gatA*, *acnB*, CD1536 and *cstA*) (Figures 2 and 3). However, the concentration of CcpA required to obtain a full shift differed depending on the target genes. To demonstrate the specificity of CcpA binding to the *cre_CD_* site-containing DNA fragments, we first showed that no shift was induced by CcpA with promoter regions of CD1893 and CD3218 or with internal DNA fragments of *feoA* and *gatA* that do not contain a *cre_CD_* site (Figures 2D and 3C). Moreover, the formation of the CcpA–DNA complex was prevented by the addition in excess of unlabelled DNA fragments containing the *cre_CD_* site of *acnB*, CD1893 and CD3218 used as a competitor (data not shown). These results validated the binding of CcpA to the targets identified by ChIP-on-Chip, including those with low-enrichment factors like *spo0A* or *spoVS*, and showed the specific interaction of CcpA with *cre_CD_* sites*.*

To further demonstrate that the *cre_CD_* site alone is required for the binding of CcpA, we performed EMSAs using DNA fragments deleted or not for the *cre_CD_* site of the *hadA*, *acnB*, *feoA*, CD1536, *gatA* and *cstA* genes. As shown in Figure 3, the deletion of the *cre_CD_* site abolished the shift induced by CcpA for the regulatory regions tested. We confirm that CcpA can interact *in vitro* with the *cre_CD_* site-containing DNA fragments of 15 genes, and we show that this binding disappears when the *cre_CD_* site is deleted for six genes. Interestingly, CcpA interacts *in vivo* with *cre_CD_* sites that are located inside of operons, and we clearly demonstrate that in the case of *acnB*, this *cre_CD_* sequence is recognized and bound by CcpA *in vivo* (ChIP-on-Chip) and *in vitro* (EMSA) leading to the strong repression of the *aksA-acnB-icd* operon by CcpA. This site internal to an operon is functional as previously shown for the *iol* operon of *B. subtilis* (51).

### Identification of additional candidates for a direct CcpA control

Using the RegPredict web server (43), we extended the search of the *cre_CD_* motif to the complete *C. difficile* genome of strain 630. We detected 901 putative *cre_CD_* sites corresponding to 783 genes with a score >4, located between position −300 to +300 from the translational start sites (Supplementary Table S3). This *in silico* analysis allowed us to identify 143 putative *cre_CD_* sites corresponding to about half of the CcpA-controlled genes in transcriptome (Supplementary Table S2). Most of these sites are adjacent or inside genes involved in carbon and energy metabolisms (35 genes), in amino acids biosynthesis or degradation (31 genes), in sporulation (7 genes), in regulation (10 genes), in cell envelope (10 genes) or encoding proteins of unknown function (29 genes).

**Figure 4.**
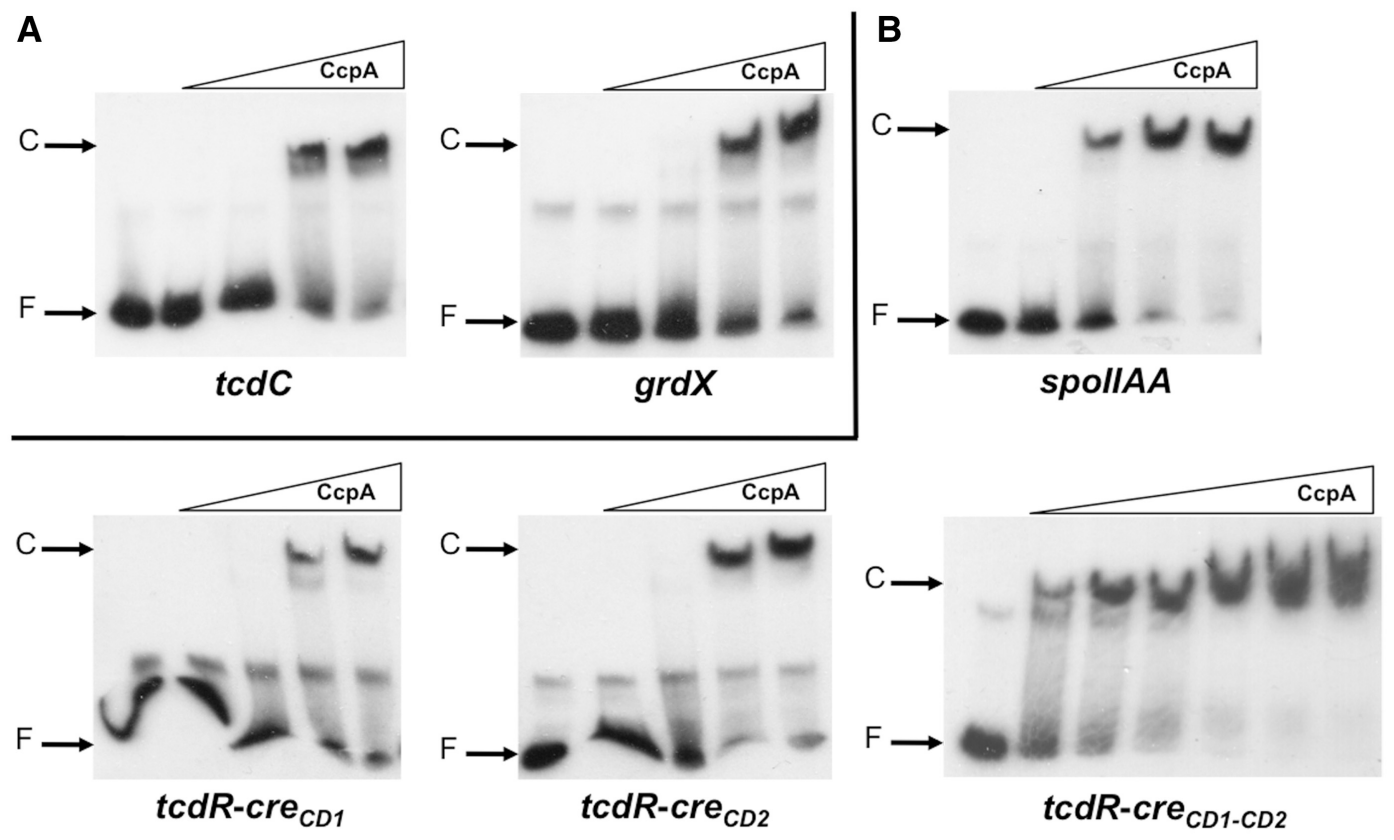
Binding of CcpA to the *cre_CD_* sites of *tcdC*, *grdX, tcdR* and *spoIIAA* target genes. EMSAs of DNA fragments containing the *cre_CD_* sites of the *tcdC* (CD0664), *grdX* (CD2357), *tcdR* (CD0659) and *spoIIAA* (CD0770) genes. The ^32^P-labelled DNA fragments were incubated with increasing concentrations of purified CcpA–His_6_ as described in the ‘Materials and Methods’ section: (**A**) 0, 32, 65, 97 and 129 nM of CcpA–His_6_ for *tcdC* and *grdX*; (**B**) 0, 129, 259, 388 and 518 nM of CcpA–His_6_ for *tcdR-cre_CD1_*,*tcdR-cre_CD2_* and *spoIIAA* and 0, 65, 97, 129, 259, 388 and 518 nM of CcpA–His_6_ for *tcdR-cre_CD1-CD2_*. The autoradiograms of the gels are shown.

To show whether at least some of these predicted *cre_CD_* sites are functional, we performed EMSAs with *cre_CD_* site-containing DNA fragments of two genes (*spoIIAA* and *grdX*) negatively regulated by CcpA in transcriptome, but not retained by CcpA in our ChIP-on-Chip experiment conditions (Supplementary Table S2). A shift of a DNA fragment containing the *cre_CD_* motif at position +198 relative to the translational start site of *grdX* was observed in the presence of 97 nM of CcpA (Figure 4A). This interaction disappeared in the presence of an excess of the same unlabelled DNA fragment (data not shown). For the *spoIIAA* gene, two different *cre_CD_* sites located at position +87 and +194 from the translational start site were found with RegPredict. We showed that 388 nM of CcpA induced a shift with the DNA fragment containing the site at position +87 (Figure 4B) and not with a DNA fragment containing the site at position +194 (data not shown). However, when we performed EMSA with a DNA fragment containing both *cre_CD_* sites, 97 nM of CcpA was sufficient to induce a shift (data not shown). These results indicated that the binding affinity of CcpA to the *spoIIAA* regulatory region increased when both *cre_CD_* sequences are present. Thus, despite the fact that we did not see the binding of CcpA to the *spoIIAA and grdX* genes in the ChIP-on-Chip conditions used, these genes could also be direct physiological CcpA targets. This suggests that CcpA might directly control additional genes with *cre_CD_* motifs deduced from the *in silico* search.

Interestingly, five putative *cre_CD_* sequences are detected in the PaLoc as follows: upstream of the translational start site of *tcdR* (positions −235 and −169) or *tcdB* (position −211) and inside the *tcdA* gene (position +277) or *tcdC* gene (position +58). The binding of CcpA to the promoter region of *tcdB* and to the 5′ end of the *tcdA* gene, which has been already demonstrated (30), is consistent with the position of the *cre_CD_* motif. Thus, we tested the possible interaction of CcpA with the *cre_CD_* sites of the promoter region of *tcdR* and of the 5′ end of the *tcdC* gene. We detected a shift with the *cre_CD_* site-containing fragment of *tcdC* in the presence of 97 nM of CcpA (Figure 4A)*.* We also observed the formation of a protein–DNA complex with a DNA fragment containing the two putative *cre_CD_* sites of *tcdR* when we used 65 nM of CcpA, whereas 388 nM was needed for a shift of the DNA fragment containing only one of each site (Figure 4B) suggesting a cooperative effect of the *cre_CD_* sites. Therefore, with the exception of *tcdE*, CcpA interacts with the promoter region or with the 5′ end of the PaLoc genes. These results show the complex regulation of toxin synthesis in response to glucose availability mediated by CcpA.

### Role of glucose and CcpA in the control of *C. difficile* carbohydrate metabolism

In this work, we used genome-wide transcriptome, ChIP-on-Chip experiments and *in silico* analysis to characterize the CcpA regulon. We discussed later in the text in more details the involvement of glucose and CcpA in the global control of key metabolic pathways and cellular processes of *C. difficile*.

**Figure 5.**
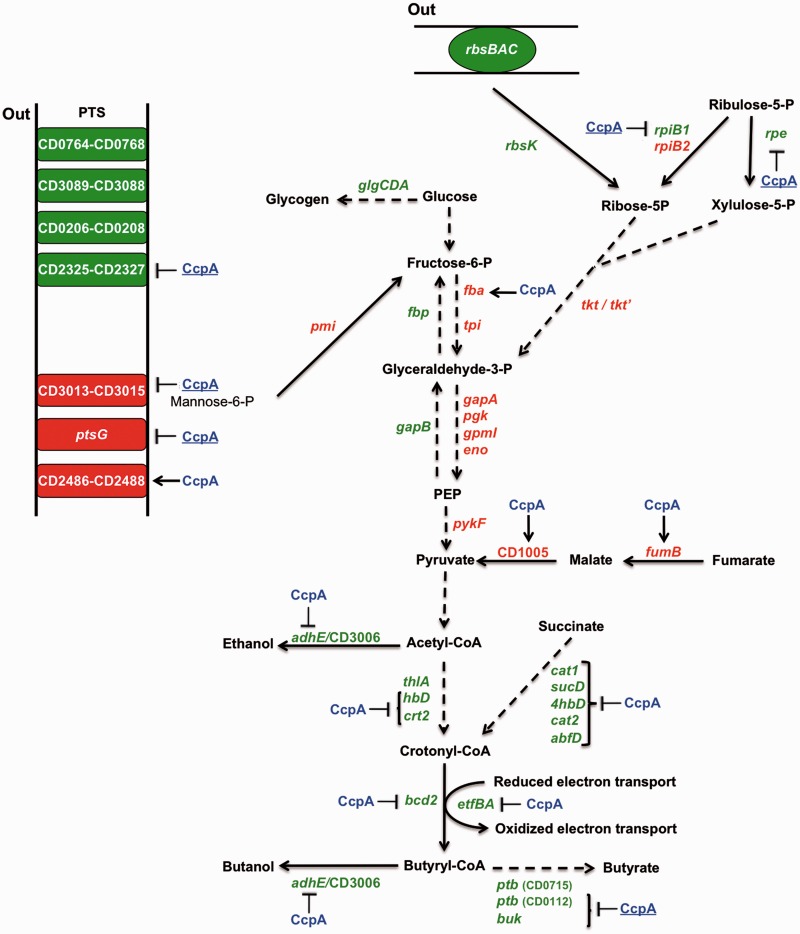
Role of glucose and CcpA in the regulation of carbohydrate utilization, central carbon metabolism and fermentation. Genes encoding enzymes or transporters, which are regulated by CcpA and/or glucose in transcriptome. Green: gene downregulated by glucose; red: gene upregulated by glucose; black: gene not regulated by glucose; blue CcpA: regulation by glucose through CcpA; blue and underlined CcpA: other type of control by CcpA; perpendicular: gene downregulated by CcpA; arrow: gene upregulated by CcpA. *glgCDA* (CD0882–CD0884), glycogen biosynthesis; *fbp* (CD1191), fructose-1,6-bisphosphatase; *fba* (CD0403), fructose-1,6-bisphosphate aldolase; *tpi* (CD3172), triosephosphate isomerase; *gapA* (CD3174) *and gapB* (CD1767), glyceraldehyde-3-phosphate dehydrogenase; *pgk* (CD3173), phosphoglycerate kinase; *gpmI* (CD3171), 2,3-bisphosphoglycerate-mutase; *eno* (CD3170), enolase; *pykF* (CD3394), pyruvate kinase; *fumB* (CD1004), fumarate hydratase subunit B; CD1005, putative nicotinamide adenine dinucleotide-dependent malic enzyme; *rpe* (CD2319), putative ribulose-phosphate 3-epimerase; *rpiB1* (CD2320), ribose-5-phosphate isomerase B1; *rpiB2* (CD3480), ribose-5-phosphate isomerase B2; *rbsK* (CD0299), ribokinase; *tkt’* (CD2321), transketolase, central and C-terminal; *tkt* (CD2322), transketolase, N-terminal; CD0764–CD0768, PTS sorbitol IIC, IIB, IIA; CD3089–CD3088, PTS cellobiose IIBC; CD2327–CD2325, PTS galactitol IIA, IIB, IIC; CD0208–CD0206, PTS fructose-like IIA, IIB, IIC; CD2486–CD2488, PTS fructose-like IIC, IIB, IIA; CD3015–CD3013, PTS mannose-specific IIA, IIB, IIC; *ptsG* (CD2667, CD2666), PTS glucose-specific IIA, IICB; *pmi* (CD2491), mannose-6-phosphate isomerase; *rbsBAC* (CD0300–CD0302), ribose ABC transporter; *adhE* (CD2966) and CD3006, aldehyde-alcohol dehydrogenase; *thlA* (CD1059), acetyl-CoA acetyltransferase; *hbd* (CD1058), 3-hydroxybutyryl-CoA dehydrogenase; *crt2* (CD1057), 3-hydroxybutyryl-CoA dehydratase; *cat1* (CD2343), succinyl-CoA: coenzyme A transferase; *sucD* (CD2342), succinate-semialdehyde dehydrogenase; *4hbd* (CD2338), 4-hydroxybutyrate dehydrogenase; *cat2* (CD2339), 4-hydroxybutyrate CoA transferase; *abfD* (CD2341), vinylacetyl-coa-δ-isomerase; *bcd2* (CD1054), butyryl-CoA dehydrogenase; *etfBA* (CD1055–CD1056), electron transfer flavoproteins; *ptb* (CD0715, CD0112), phosphate butyryltransferase; *buk* (CD0113), butyrate kinase.

Compared with other pathogenic clostridia, *C. difficile* has a larger repertoire of EII PTS (51 PTS in strain 630), and this bacterium is able to ferment several sugars taken up by PTS, such as fructose, glucose, mannitol and mannose (52). In the presence of glucose, we found that *C. difficile* preferentially expresses glucose (CD2667–CD2666), fructose (CD2486–CD2488) and mannose (CD3015–CD3013) PTS, whereas the synthesis of some PTS of secondary carbon sources, such as sorbitol (CD0764–CD0768), galactitol (CD2327–CD2325) or cellobiose (CD3089–CD3088), was repressed by glucose (Figure 5). All PTS transporters induced by glucose were regulated by CcpA. Among the PTS systems repressed by glucose, only the galactitol PTS was controlled by CcpA. We further demonstrated by qRT-PCR that during exponential growth, the expression of the *gatABC* operon was strongly repressed by glucose through CcpA (Supplementary Figure S2). The expression of the *rbsRKBAC* (CD0298–CD0302) operon encoding the ribose repressor, a ribose ABC transporter and the ribokinase was repressed by glucose in *C. difficile*. Moreover, when we looked at the *rbsR* expression at mid-exponential growth phase by qRT-PCR analysis, we observed that *rbsR* expression was partially repressed by CcpA in response to glucose (Supplementary Figure S2). We demonstrated in ChIP-on-Chip experiment that the *rbs* operon and genes encoding cellobiose or galactitol PTS (CD2884-CD2880 and CD2327-CD2325) and two other PTS systems (CD0467–CD0469 and CD0490–CD0494 operons) were direct CcpA targets (Table 2 and Figure 3A). Only a few PTS and non-PTS sugar utilization systems were regulated by glucose and/or CcpA in transcriptome in the conditions we used. However, other genes involved in sugar assimilation systems were bound by CcpA in ChIP-on-Chip. We cannot exclude that genes involved in carbohydrate utilization are not detected in transcriptome, as they are expressed at basal level in the absence of sugar inducers.

After sugar uptake, the carbohydrates are metabolized through the central carbon metabolism to produce energy and precursors for anabolism. Glucose addition regulates several pathways of the central carbon metabolism, such as glycolysis, gluconeogenesis and pentose-phosphate pathway (PPP) in *C. difficile*. The expression of *fba* encoding the fructose-1,6-bisphosphate aldolase, a key enzyme of glycolysis, was strongly induced by glucose and positively regulated by CcpA (Figure 5). However, most genes encoding for the central glycolysis/gluconeogenesis pathway were regulated by glucose independently of CcpA. In fact, the *gapA-pgk-tpi-gpmI-eno* operon (CD3174–CD3170) was strongly induced by glucose, whereas the expression of *gapB* (CD1767) and *fbp* (CD1191) was repressed. Likewise, in *B. subtilis*, the expression of the *gapA* operon is induced by glucose, whereas the *gapB* and *pckA* genes involved in gluconeogenesis are repressed by glucose through a CcpA-independent mechanism (18). The PPP is an alternative route of glucose degradation that generates nicotinamide adenine dinucleotide phosphate (NADPH) and pentoses (Figure 5). Among the genes involved in this pathway, synthesis of the transketolase from the *tkt*’ and *tkt* genes (CD2321, CD2322) and the ribose-5-P isomerase from *rpiB2* (CD3480) was induced by glucose in strain JIR8094 (Figure 5). In *B. subtilis* and *S. aureus*, only the gluconate conversion enzymes of the PPP to provide ribulose 5-P are weakly induced by glucose (18,46).

Glucose and/or CcpA regulate the synthesis of several other enzymes involved in carbon metabolism. The *aksA-acnB-icd* operon (CD0832–CD0834), which encodes a *trans*-homoaconitate synthase, an aconitate hydratase and an isocitrate/3-isopropylmalate dehydrogenase, respectively, was strongly repressed by glucose through CcpA. As mentionned previously, we found a *cre_CD_* site inside *acnB*, and we showed that it is a direct CcpA target *in vivo* (Table 2) and *in vitro* (Figure 3B). Moreover, expression of *fumB* (CD1004) involved in malate formation from fumarate and of a gene encoding a protein sharing 57% identity with YtsJ of *B. subtilis* involved in malate to pyruvate interconversion (CD1005) was induced by glucose in a CcpA-dependent manner (Figure 5, Supplementary Table S2). However, these genes lack *cre_CD_* sites, therefore, suggesting an indirect effect of CcpA. Finally, the pyruvate carboxylase *pycA* gene (CD0021) leading to oxaloacetate production from pyruvate was induced in the presence of glucose by a CcpA-independent mechanism.

### The role of glucose and CcpA in the regulation of energy metabolism

The principal source of energy in *C. difficile* comes from the catabolism of carbohydrates and amino acids. *Clostridium difficile* metabolizes carbohydrates through fermentation, leading to the production of short-chain fatty acids (lactate, acetate, butyrate, propionate … ) and minor quantity of alcohols (12,53). The pathways allowing the synthesis of butyryl-CoA from acetyl-CoA (CD1054–CD1059) or succinate (CD2344–CD2338) and the further production of butyrate (CD0112–CD0113) or butanol (CD2966) from butyryl-CoA were repressed by glucose through a CcpA-dependent mechanism (Figure 5, Supplementary Tables S2 and S4). Among these genes, *cre_CD_* sites are present inside the *bcd2* operon (CD1054–CD1059) and upstream of CD2344 and *adhE* (CD2966) (Figure 5 and Supplementary Table S2).

**Figure 6.**
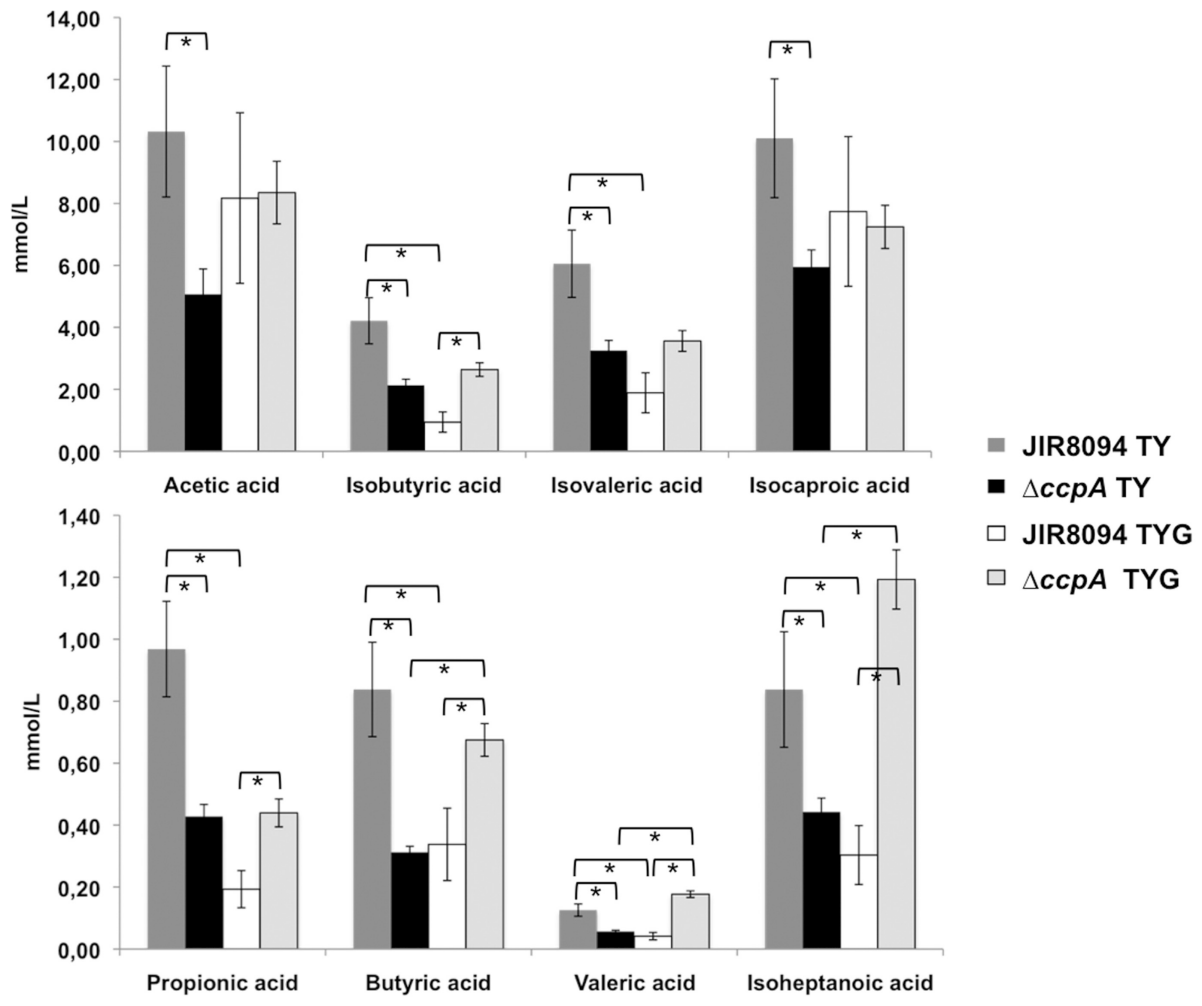
Fermentation end products of the strain JIR8094 and the *ccpA* mutant grown in the presence or absence of glucose. Gas–liquid chromatography analysis of the metabolic end products from JIR8094 and *ΔccpA* mutant strains after 48 h of growth in TY or TYG medium was performed. The mean and standard error of three experiments are shown. Asterisk corresponds to significative statistical difference analysed by the student *t*-test (*P* < 0.05).

To confirm the impact of glucose addition and CcpA regulation on the fermentation processes, we analysed by gas–liquid chromatography the end products of fermentation of strain JIR8094 and the *ccpA* mutant grown in TY or in TYG. We could not detect butanol nor ethanol by these assays. The formation of butyric, isobutyric, valeric, propionic and isoheptanoic acids was repressed by glucose, and this downregulation was fully or partially abolished in a *ccpA* mutant in TYG (Figure 6). These results are in agreement with our transcriptome data, indicating that key steps of fermentation are repressed by glucose through CcpA. It is intriguing to note that the *ccpA* gene disruption led to a decreased production of the end products of fermentation in the absence of glucose. This effect could result from a change in the metabolism balance because of the lack of CcpA, to an indirect influence of CcpA in the fermentation pathways through the modulation of intracellular concentration of metabolites or a cascade of regulation by uncharacterized mechanisms.

*Clostridium difficile* can also use amino acids as energy sources through Stickland reactions. These reactions consist in the coupled fermentation of two amino acids, in which one is oxidatively deaminated or decarboxylated (Stickland donor) and another is reductively deaminated or reduced (Stickland acceptor). The most efficient Stickland donors are valine, leucine, isoleucine and alanine, whereas the most efficient acceptors are proline, glycine and leucine (54,55).

**Figure 7.**
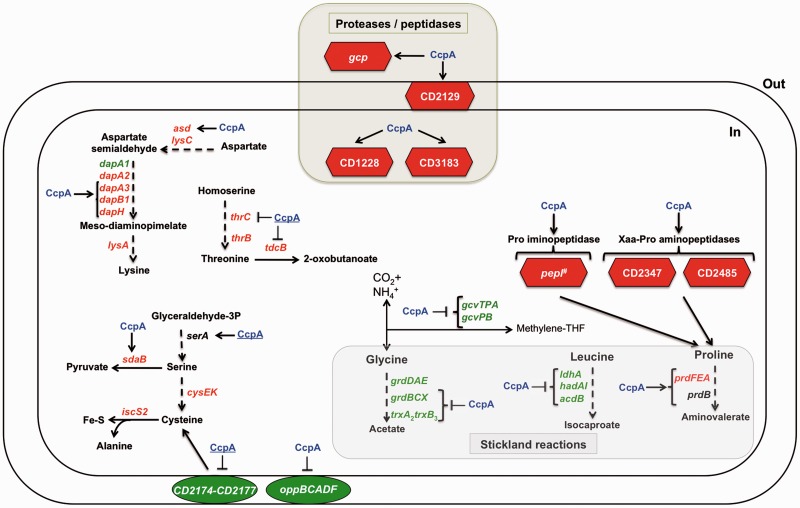
Role of glucose and CcpA in the control of peptide and amino acid metabolisms. Genes encoding enzymes or transporters, which are regulated by CcpA and/or glucose in transcriptome. Green: gene downregulated by glucose; red: gene upregulated by glucose; black: gene not regulated by glucose; blue CcpA: regulation by glucose through CcpA; blue and underlined CcpA: other type of control by CcpA; hash: localization unknown; perpendicular: gene downregulated by CcpA; arrow: gene upregulated by CcpA. *lysC* (CD2054), aspartokinase; *asd* (CD3224), aspartate-semialdehyde dehydrogenase; *dapA1* (CD3000), dihydrodipicolinate synthase 1; *dapA2* (CD3223), dihydrodipicolinate synthase 2; *dapA3* (CD3225), dihydrodipicolinate synthase 3; *dapB1* (CD3226), dihydrodipicolinate reductase; *dapH* (CD3227), 2,3,4,5-tetrahydropyridine-2,6-dicarboxylate; *lysA* (CD2053), diaminopimelate decarboxylase; *thrC* (CD2118), threonine synthase, *thrB* (CD2119), homoserine kinase; *tdcB* (CD2514), threonine dehydratase II; *serA* (CD0995), putative D-3-phosphoglycerate dehydrogenase; *sdaB* (CD3222), L-serine dehydratase; *cysE* (CD1595), serine acetyltransferase; *cysK* (CD1594), *O*-acetylserine sulphydrylase; *iscS2* (CD1279), cysteine desulphurase; *oppBCADF* (CD0853–CD0857), oligopeptide ABC transporter; CD2177–CD2174, cystine ABC transporter; *prdF* (CD3237), proline racemase; *prdE* (CD3239), proline reductase; *prdA* (CD3244), D-proline reductase proprotein; *prdB* (CD3241), proline reductase; *trxB3* (CD2356), thioredoxin reductase 3; *trxA2* (CD2355), thioredoxin 2; *grdX* (CD2357), glycine reductase complex component; *grdE* (CD2354), glycine reductase complex component B subunits α and β; *grdA* (CD2352), glycine reductase complex selenoprotein A; *grdB* (CD2351), glycine reductase complex component B γ subunit; *grdC* (CD2349), glycine reductase complex component C subunit β; *grdD* (CD2348), glycine reductase complex component C subunit α; *ldhA* (CD0394), 2-hydroxyisocaproate dehydrogenase; *hadA* (CD0395), 2-hydroxyisocaproate CoA transferase; *hadI* (CD0396), activator of dehydratase; *acdB* (CD0399), acyl-CoA dehydrogenase; *gcvTPA* (CD1657), bi-functional glycine dehydrogenase/aminomethyl transferase protein; *gcvPB* (CD1658), Glycine decarboxylase; CD1228, putative protease; CD3183, putative peptidase; CD2485, putative Xaa-Pro aminopeptidase; CD2347, putative Xaa-Pro dipeptidase; *gcp* (CD0152), putative O-sialoglycoprotein endopeptidase; CD2129, putative membrane-associated peptidase; *pepI* (CD3041), proline iminopeptidase.

The expression of *prdE*, *prdF* and *prdA* genes (CD3237, CD3239 and CD3244), required for proline reduction, was induced by glucose and positively controlled by CcpA (Figure 7, Supplementary Table S2 and Figure S2). Actually, *prdA* harbours a *cre_CD_* site in the promoter region to which CcpA was able to bind *in vivo* and *in vitro* (Table 2 and Figure 2C). The expression of *ldhA*, *hadA*, *hadI*, *acdB* and *etfB1* genes (CD0394, CD0395, CD0396, CD0399 and CD0400) required for the leucine reductive branch leading to the formation of isocaproate was 4-fold repressed by glucose by a CcpA-dependent mechanism (Figure 7). We also identified *hadA* as a CcpA direct target by ChIP-on-Chip analysis (Table 2) and demonstrated that the *cre_CD_* site located upstream of *hadA* was essential for CcpA binding, as its deletion no longer showed a shift by EMSA analysis (Figure 3A). Finally, most of the genes of the *trxA2-trxB3-grdXEABCD* operon involved in glycine reduction (CD2358–CD2348) and *gcv* operon involved in glycine oxidation (CD1657–CD1658) were repressed by CcpA in reponse to glucose (Figure 7) (56). Moreover, CcpA binds to the *cre_CD_* motif internal to *grdX* (CD2357) (Figure 4A) and a *cre_CD_* site is present upstream of *gcvPB* (CD1658) (Table 2). Despite the fact that the utilization of serine to produce energy has not been described in *C. difficile*, this amino acid is preferentially degraded with threonine, arginine, cysteine and proline by *Clostridium sticklandii* that is closely related to *C. difficile.* Interestingly, the expression of *sdaB* (CD3222) involved in serine degradation to form pyruvate that can be further metabolized by fermentation was induced by glucose and positively controlled by CcpA (Figure 7).

Thus, CcpA seems to play a key role in the hierarchy of amino acid utilization in *C. difficile*. It directly regulates the genes involved in Stickland reactions by using preferentially proline as an energy source in detriment of leucine or glycine. It is worth adding that addition of proline represses toxin synthesis, whereas genes encoding enzymes for the reductive pathway of glycine and leucine are upregulated during growth conditions that favour toxin production (57). This observation suggests a connection between proline utilization and the control of toxin synthesis that probably involves a regulation by CcpA.

### Amino acids metabolism

In *C. difficile*, the synthesis of several proteases and peptidases was regulated by CcpA in response to glucose (Figure 7). Among them, the expression of *gcp* (CD0152) encoding an *O*-sialoglycoprotein endopeptidase and three other peptidases encoding genes (CD1228, CD2129 and CD3183) was induced by glucose in a CcpA-dependent manner. Similarly, the same type of regulation was found for the proline iminopeptidase gene *pepI* (CD3041) and peptidases encoding genes (CD2347 and CD2485) that share similarities with Xaa-Pro dipeptidases. So, we observed a coordinated regulation of the peptidases (release of free proline) and the proline reductase encoding genes (54). By contrast, the expression of the *oppBCADF* operon (CD0853–CD0857) encoding an oligopeptides ABC transporter was repressed by glucose through a CcpA-dependent mechanism. Furthermore, CD2347 and *oppBCADF* operons are CcpA direct targets bound by CcpA *in vivo* and harbouring a *cre_CD_* site (Table 2).

In *C. difficile*, the glucose induces the biosynthetic pathways of lysine, threonine, serine and cysteine, and CcpA plays a role in the control of some of them (Figure 7, Supplementary Table S2). The expression of *asd-dapA3-dapB1* and *dapH* (CD3224–CD3227) involved in lysine biosynthesis from aspartate was positively controlled by CcpA in response to glucose availability (Figure 7). Expression of the *cysK-cysE* (CD1594–CD1595) and the *iscR-iscS2-iscU* (CD1278–CD1280) operons involved in cysteine synthesis and iron–sulphur biogenesis from cysteine (sulphur donor), respectively, was increased in the presence of glucose independently of CcpA (Figure 7, Supplementary Table S2). Thus, in the presence of glucose, the increased expression of pathways required for iron-sulphur production might be related to the crucial role of iron–sulphur proteins in electron transfer and fermentation pathways (58). In conclusion, we show that CcpA in *C. difficile* is not only involved in the control of carbon metabolism but is also implicated in the regulation of nitrogen metabolism as described for *B. subtilis* (17).

### Control of stress response and cell wall metabolism by glucose and CcpA

During host infection, *C. difficile* is exposed to several stresses and responds by inducing different mechanisms of defense, such as the synthesis of the heat shock proteins (Hsps), which are also induced by acid and oxidative stresses in *C. difficile* (59). We observed that CcpA regulated the expression of genes involved in heat stress response in the presence of glucose likely through an indirect control, as we did not detect *cre*_CD_ sites upstream of these genes (Supplementary Table S2). The expression of certain genes implicated in oxidative stress, such as *norV*, encoding a protein involved in nitric oxide (NO) detoxification, and *trxB* (CD1691), encoding a thioredoxin reductase was induced by glucose through CcpA. Furthermore, the presence of a *cre_CD_* site in *norV* (CD1157) indicated that this gene is probably directly regulated by CcpA (Supplementary Table S2). The involvement of CcpA in the regulation of stress proteins has also been reported in *Lactobacillus plantarum* (60) and in *Enterococcus faecalis* (61).

Previous studies showed the involvement of cell surface-associated proteins in *C. difficile* colonization (5). We observed that CcpA in response to glucose or CcpA alone was implicated in the control of a large number of genes encoding cell wall proteins and peptidoglycan hydrolases potentially implicated in *C. difficile* adhesion to the host cells (Supplementary Table S2). Moreover, a *cre_CD_* site is present in the *dltDABC* operon that was repressed by CcpA (Supplementary Table S2). This operon encodes for proteins responsible for the teichoic acids D-alanylation, playing an important role in the bacterial autolytic activity (62). In *S. pneumoniae*, CcpA has also been reported to be implicated in the regulation of genes encoding for cell surface proteins important for colonization (23).

### Involvement of glucose and CcpA in sporulation

It has been recently described that CcpA is one of the few regulators present in the proteome of *C. difficile* spores (63). Sporulation and germination are key events of the infectious cycle of *C. difficile* (31), and spores mainly contribute to nosocomial transmission. In *C. difficile,* the global response regulator Spo0A (CD1214) and the associated sensor histidine kinase CD1579 (64) are involved in the sporulation initiation. We observed from the transcriptome that CcpA negatively regulated *spo0A* and CD1579 expression in the presence of glucose (Supplementary Table S2). By qRT-PCR, we further demonstrated that *spo0A* was also 2–3-fold repressed by glucose (Supplementary Table S4). Moreover, the expression of several genes involved in different stages of sporulation, such as *sigE* (CD2643) and *sigG* (CD2642) and *spoIIAA-spoIIAB-sigF* (CD0770–CD0772), *spoIIE* (CD3490), *spoIIIAG* (CD1198), *spoIVA* (CD2629) and *spoVS* (CD1935), was repressed by glucose in the absence of CcpA. CcpA also negatively regulated most of these *spo* genes in the absence of glucose (Supplementary Table S2). Furthermore, we found that the *spo0A* gene encoding the key regulator of the initiation of sporulation and the *spoIIAA-spoIIAB-sigF* operon, which is responsible for the synthesis and the activation/inactivation of SigF (involved in the endospore formation), are direct targets that contain *cre_CD_* sites (Table 2) to which CcpA is able to bind (Figures 2 and 4B).

**Figure 8.**
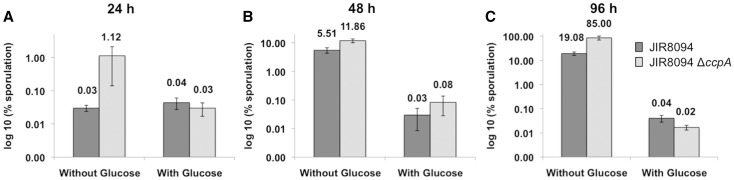
Effect of glucose addition or CcpA inactivation on the sporulation efficiency. Comparison of sporulation efficiency of *C. difficile* JIR8094 and *ccpA* mutant strains grown in SM medium supplemented or not supplemented with 0.5% of glucose at 24 h (**A**), 48 h (**B**) and 96 h (**C**). Total cell counts were obtained by enumeration of CFUs derived from serial dilutions of the initial culture plated on BHI agar supplemented with 0.1% of taurocholate. The same procedure was applied for spore counts with an additional step of heat treatment at 60°C for 30 min, to select for heat resistant spores. Percentage of sporulation is the ratio between the spore titer and total cell titer at each time point. The mean and standard error of three experiments are shown.

We further tested the involvement of glucose and CcpA inactivation on the sporulation efficiency during 96 h of growth (Figure 8). After 48 h of growth, we observed that the sporulation efficiency of JIR8094 and *ccpA* mutant strains was similar and strongly reduced when glucose was added. This indicates that sporulation is strongly repressed by glucose and that this repression does not involve CcpA. This has already been reported for *C. perfringens* where glucose-mediated catabolite repression of sporulation is not because of CcpA activity (26). Therefore, like in *C. perfringens*, glucose-repression of *C. difficile* sporulation implicates a still uncharacterized CcpA-independent pathway. In addition*,* CcpA is required for sporulation in *C. perfringens* in the absence of glucose, with a 60-fold drop in sporulation efficiency in a *ccpA* mutant at 24 h of growth in DSSM medium (26). On the contrary, in *C. difficile*, the rate of sporulation efficiency in the absence of glucose was 8–37-fold higher in the *ccpA* mutant compared with the JIR8094 strain at 24 h of growth in SM medium (Figure 8A). This is in agreement with the transcriptome results showing that CcpA is implicated in the repression of genes involved in the control of the first stages of sporulation like the SpoIIAA operon and the SpoIIE phosphatase (Supplementary Table S2).

### Complex regulatory network mediated by CcpA

There are few studies concerning the combination of regulators that control gene expression during host infection. The data generated herein demonstrated that glucose and/or CcpA controlled the expression of 67 regulators (Supplementary Table S2). Twenty of them were regulated by glucose through a CcpA-dependent mechanism. We deduced from the transcriptome analysis that CcpA controlled 30 regulators, and we revealed by ChIP-on-Chip experiments that five transcriptional regulators (*rbsR*, *cggR*, CD1584, CD2111 and *spo0A*) and a sensor histidine–kinase (*virS*) were direct CcpA targets (Table 2). However, regulation of expression of genes encoding the DeoR type regulator CD2111 and VirS was not observed in the transcriptome conditions used, and this may be because of the low level of their expression. In addition, the CcpA-controlled regulatory genes CD1428 and CD3031 contain a *cre_CD_* site (Supplementary Table S2). We suggest that these seven CcpA direct targets may mediate through a cascade of regulation part of the indirect regulation by CcpA observed in our analysis. Finally, CcpA indirectly controlled several additional regulators (Supplementary Table S2). These regulators might be under the control of direct CcpA regulators or their activity might be modulated by other mechanisms, including changes in the availability of metabolic effectors as proposed for CggR and CcpC in *B. subtilis* (65). Interestingly, in *B. subtilis*, a *cre* sequence is located upstream of *cggR*, but *cggR* transcription is affected by a *ccpA* deletion independently of this *cre* (65,66).

The ability of CcpA to promote different regulatory outcomes, leading to activation or repression, suggests that additional modulating signals and/or regulatory pathways might interact with CcpA in the regulation of specific target genes. CodY is a pleiotropic transcriptional regulator that monitors the nutrient sufficiency of the environment. In *C. difficile*, CodY regulates the toxin genes, and genes implicated in amino acids biosynthesis, nutrient transport, fermentation and sporulation (67). Several genes are found directly regulated by CcpA and CodY. It is the case of toxin specific regulator gene *tcdR*, the *oppBCADF* operon for oligopeptide transport, the butyrate metabolism operon (CD1054–CD1059) or genes implicated in the early stages of sporulation, such as the *spoIIAA* operon and *spoIIE* (67). This means that CcpA and CodY play a major role in several circuits of regulation in *C. difficile* and that these circuits could overlap between them rendering even more complex the regulatory network.

## CONCLUSION

In conclusion, our results show the importance of the global regulator CcpA in *C. difficile* as a key factor controlling several processes, such as selective utilization of carbon sources, differential fermentation pathways, amino acid metabolism and toxin production in response to glucose availability. The identification of CcpA targets *in vivo* and *in vitro*, and also the definition of the *C. difficile cre* consensus allow us to dissociate direct and indirect effects of CcpA. The CcpA direct targets are mainly involved in the utilization of alternative sugar sources (PTS or non-PTS sugar transporters), in fermentation pathways leading to the production of butyrate, in Stickland reactions, in toxin synthesis and in regulatory functions including two key steps of the initiation of sporulation.

Phylogeny analysis shows that CcpA of *C. difficile* clusters with that of clostridia, but not with CcpA of bacilli (30). Interestingly, we found putative *cre_CD_*-like sites in the regulatory regions of genes known to be controlled by CcpA in other clostridia, including the xylose utilization gene *xylB* in *C. acetobutylicum* (22) and the perfringolysin O gene *pfoA* in *C. perfringens* (29). A comparative genomic analysis of distribution of CcpA binding sites in bacteria from the bacilli and clostridia groups reinforces these observations on the divergence of the *cre* motifs in these two phylogenetically distant groups of Firmicutes and the relatively good conservation of the *cre* motifs inside of the clostridia and bacilli groups (D.A.R and N.V.S, unpublished observation). The characterization of a *cre* consensus motif for clostridia will probably help for the determination of the CcpA regulons, which remain poorly characterized in clostridia compared with other Firmicutes.

The high number of regulators controlled by CcpA either directly or indirectly suggests that CcpA plays a central role in the hierarchical organization of the genetic regulatory network of *C. difficile* as shown for *B. subtilis* (68). We are also aware that *C. difficile* virulence depends on coordinate interactions between multiple regulatory circuits. As an example, CodY has been shown to have a direct role in the regulation of sporulation and of toxin genes (67). The next step will be to understand how CcpA and CodY, two major global regulators of metabolism, interact to coordinate virulence in response to environmental signals. In *S. aureus*, the trichloroacetic acid cycle seems to be intimately integrated into regulating virulence factor synthesis and biofilm formation through specific or global regulators like CodY and CcpA (69). In *C. difficile*, we might propose that the fermentation processes are similarly correlated with the signal transduction pathways controlling virulence. Indeed, addition of butyric acid induces toxin synthesis, and during high-toxin synthesis, genes coding for the enzymes involved in the formation of butyrate from succinate or acetyl-CoA are upregulated (53,57). CodY and CcpA are implicated in the regulation of genes involved in these fermentation pathways and in toxin production linking these two processes. Finally, it is possible that CcpA might also contribute to the ability of this pathogen to obtain key nutrients in the host environment and to colonize the gut. Experiments are now in progress to further characterize the role of CcpA and its regulon during growth and survival in the animal models of CDI.

## SUPPLEMENTARY DATA

Supplementary Data are available at NAR Online: Supplementary Tables 1–4 and Supplementary Figures 1 and 2.

## FUNDING

Research grant US Public Health Service [AI057637]; predoctoral fellowship Fundação para a Ciência e a Tecnologia [SFRH//BD/16399/2004 to A.A.] and ERA-NET PathoGenoMics/CDIFFGEN (to A.A. and E.C.). U.S. Department of Energy [DE-SC0004999] and Russian Foundation for Basic Research [10-04-01768] (to D.A.R.). Funding for open access charge: Institut Pasteur.

*Conflict of interest statement*. None declared.

## Supplementary Material

Supplementary Data
