# Supplementary material for: Global transcriptional control by glucose and carbon regulator CcpA in Clostridium difficile
Source: Nucleic Acids Res. 2012 Sep 18;40(21):10701–18. doi: 10.1093/nar/gks864 (PMC3510511; doi:10.1093/nar/gks864)
Supplement: Supplementary Data [file supp_40_21_10701__index.html]

Global transcriptional control by glucose and carbon regulator CcpA in Clostridium difficile — Global transcriptional control by glucose and carbon regulator CcpA in Clostridium difficile — Supplementary Data 

# Global transcriptional control by glucose and carbon regulator CcpA in *Clostridium difficile*

## Supplementary Data

files

**Files in this Data Supplement:**

- Supplementary Data - pdf file
- Supplementary Data - xlsx file
- Supplementary Data - docx file
