# Supplementary material for: Global transcriptional control by glucose and carbon regulator CcpA in Clostridium difficile
Source: Nucleic Acids Res. 2012 Sep 18;40(21):10701–18. doi: 10.1093/nar/gks864 (PMC3510511; doi:10.1093/nar/gks864)
Supplement: Supplementary Data [file supp_gks864_nar-01170-x-2012-File010.pdf]

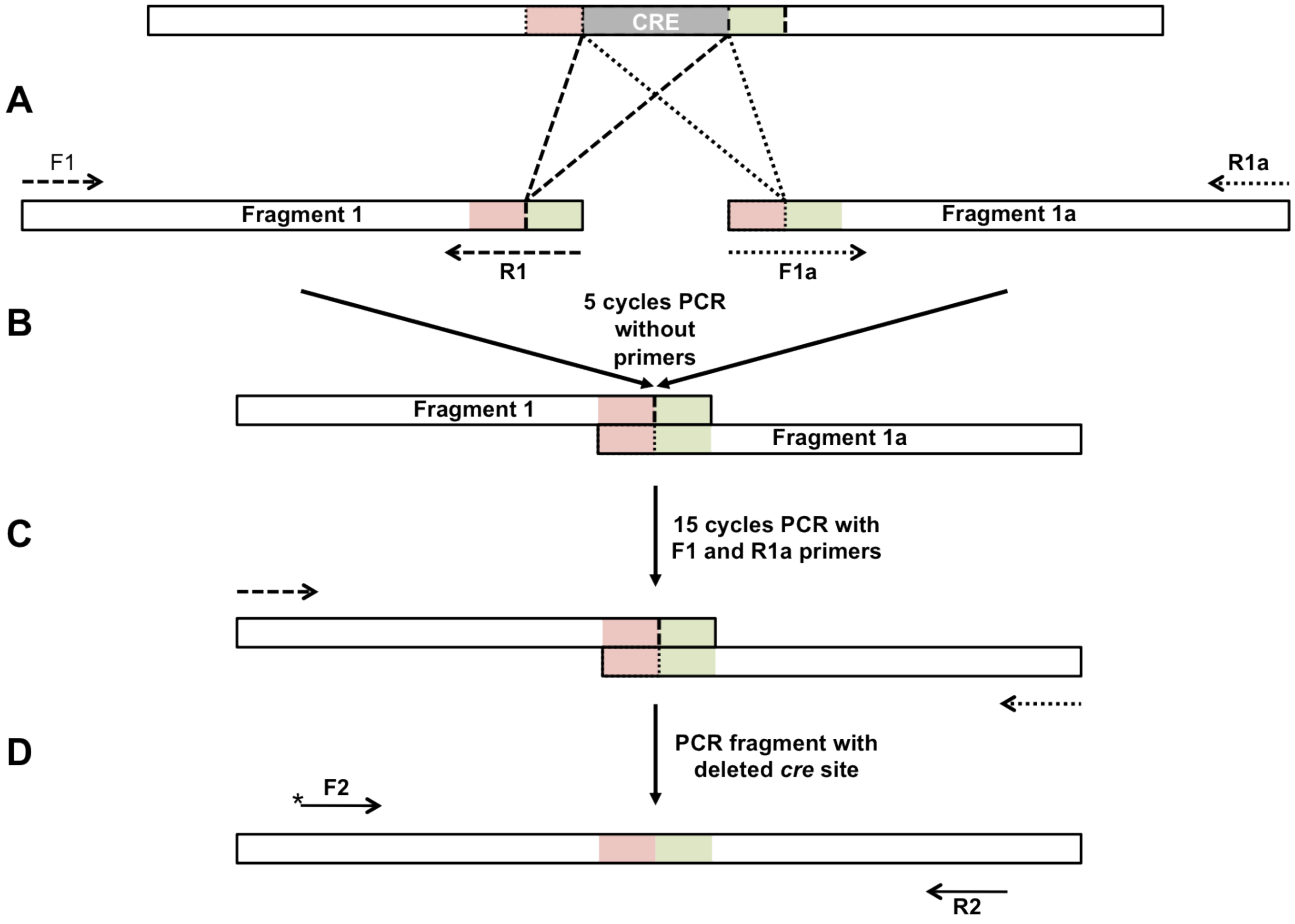

Figure S1. Methodology of the cre_{CD} motif deletion by self-priming PCR.

(A) Amplification of the fragments 1 and 1a using mutated primers R1 and F1a permitting the deletion of cre_{CD} site. (**B**) PCR of 5 cycles without primers in order to hybridize the fragments 1 and 1a in the overlap regions. (**C**) PCR of 15 cycles with external primers F1 and R1a permitting the amplification of the deleted cre_{CD} site DNA fragment. (**D**). PCR of 15 cycles with γ^{32}P-ATP radiolabelled F2 primer and R2 primer from deleted cre_{CD} site fragment.

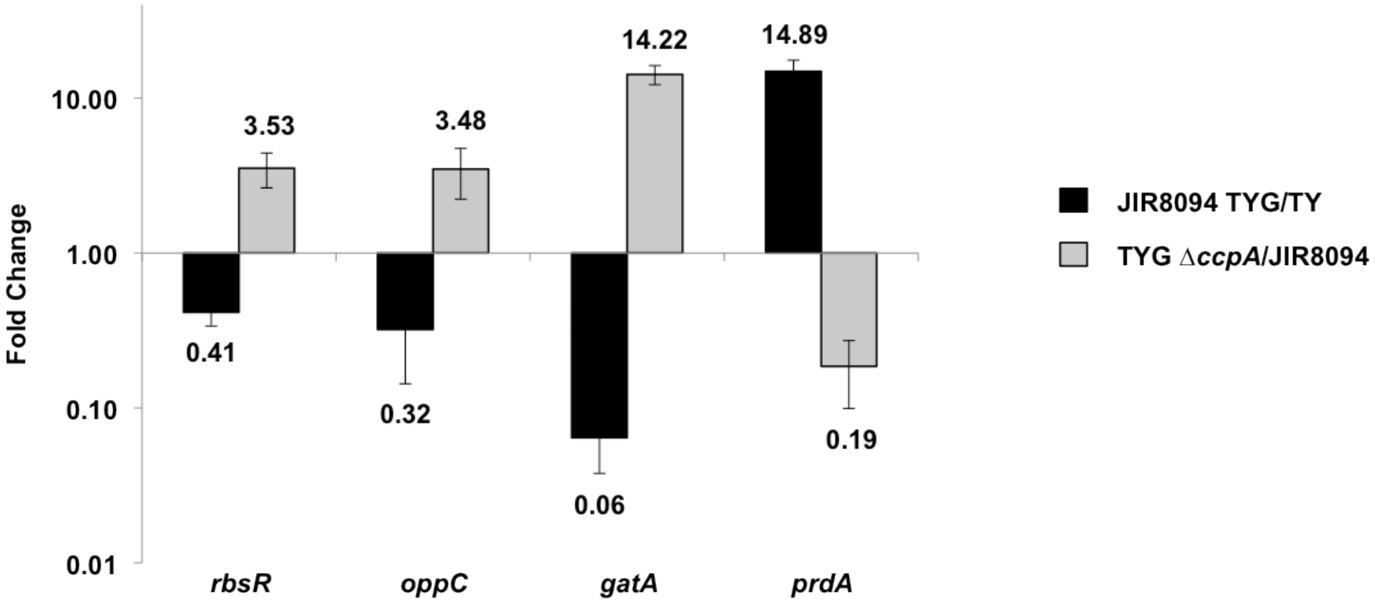

Figure S2. Effect of glucose and CcpA on the transcription of *rbsR*, *oppC*, *gatA* and *prdA* during exponential growth phase.

Relative expression of *rbsR*, *oppC*, *gatA* and *prdA* determined by qRT-PCR analysis. RNAs were extracted from 3 independent cultures of JIR8094 or *ccpA* mutant strains harvested during exponential growth phase at an OD_{600} of 0.6. The fold change was calculated using the $2^{(-\Delta\Delta Ct)}$ method. Gene expression was normalized using the housekeeping *dnaF* gene (CD1305). Error bars indicate standard deviation. Student's *t*-test was used to determine the significant differences (p.value < 0.05).
