# Supplementary material for: Global transcriptional control by glucose and carbon regulator CcpA in Clostridium difficile
Source: Nucleic Acids Res. 2012 Sep 18;40(21):10701–18. doi: 10.1093/nar/gks864 (PMC3510511; doi:10.1093/nar/gks864)
Supplement: Supplementary Data [file supp_gks864_nar-01170-x-2012-File012.docx]

**Table S1**. **List of oligonucleotides used in this study.**

**Table S2**. **List of genes that are differentially expressed after growth in the presence or absence of glucose and regulated by CcpA**.

The effect of glucose was analyzed in both JIR8094 strain (WT TYG/TY) and the derivate *ccpA* mutant (*ccpA* TYG/TY), and the CcpA involvement in the absence (TY *ccpA*/WT) or in the presence (TYG *ccpA*/WT) of glucose. Green: genes down-regulated with a fold change ≤ 0.5; Red: genes up-regulated with a fold change ≥ 2; Blue: significant p.value < 0.05.

**Table S3**. **List of the *cre_CD_* motifs found in the *C. difficile* genome using the RegPredict webserver.**

**Table S4.** **Validation of Microarrays data by qRT-PCR on 15 selected genes.**

^a^. Results from at least two independent experiments.

^b^. Results from four independent experiments.

green: genes significantly (p.value < 0.05) down-regulated with a fold change ≤ 0.5; red: genes significantly (p.value < 0.05) up-regulated with a fold change ≥ 2.

**Figure S1. Methodology of the *cre_CD_* motif deletion by self-priming PCR.**

**(A)** Amplification of the fragments 1 and 1a using mutated primers R1 and F1a permitting the deletion of *cre_CD_* site. (**B**) PCR of 5 cycles without primers in order to hybridize the fragments 1 and 1a in the overlap regions. (**C**) PCR of 15 cycles with external primers F1 and R1a permitting the amplification of the deleted *cre_CD_* site DNA fragment. (**D**). PCR of 15 cycles with γ^32^P-ATP radiolabelled F2 primer and R2 primer from deleted *cre_CD_* site fragment.

**Figure S2. Effect of glucose and CcpA on the transcription of *rbsR*, *oppC*, *gatA* and *prdA* during exponential growth phase.**

Relative expression of *rbsR*, *oppC*, *gatA* and *prdA* determined by qRT-PCR analysis. RNAs were extracted from 3 independent cultures of JIR8094 or *ccpA* mutant strains harvested during exponential growth phase at an OD_600_ of 0.6. The fold change was calculated using the 2^(-∆∆Ct)^ method. Gene expression was normalized using the housekeeping *dnaF* gene (CD1305). Error bars indicate standard deviation. Student’s *t*-test was used to determine the significant differences (p.value < 0.05).
